# Environmental Physiotherapy for Chronic Spinal Pain Management: An International Modified Delphi Study

**DOI:** 10.1002/ejp.70354

**Published:** 2026-08-11

**Authors:** Emmanuelle Opsommer, Natalya Korogod, Aníbal Manuel Melo Pereira

**Affiliations:** ^1^ HESAV School of Health Sciences—Vaud HES‐SO University of Applied Sciences and Arts Western Switzerland Lausanne Switzerland

## Abstract

**Background:**

Chronic spinal pain is a major public health challenge, but its management seldom considers the environmental impact of healthcare practices. This study aimed to identify consensus‐based recommendations for integrating environmental sustainability into chronic spinal pain care.

**Methods:**

Clinical practice guidelines on chronic spinal pain and literature on sustainable practices were reviewed to develop an initial set of statements. Following the ACCORD reporting guideline, a modified three‐round Delphi study was conducted to obtain international consensus on the statements' agreement and importance. Physiotherapists from clinical, educational, and research backgrounds participated. The initial domains included environmental advocacy and policies, education, research and innovation, community engagement, sustainable clinical practices. Consensus was defined as an interquartile range (IQR) ≤ 1 for both dimensions.

**Results:**

The preparatory review of clinical guidelines and sustainability literature identified five domains and informed the development of 47 statements and seven questions. Of the 150 experts invited, 49 participated (44 in Round 1, 35 in Round 2, 30 in Round 3), representing diverse clinical, educational, and research backgrounds from multiple world regions. By the end of the three rounds, 49 statements were classified as consensus recommendations; items with a median of 3 were reported separately as uncertain statements.

**Conclusions:**

This international Delphi study achieved broad consensus on integrating sustainable physiotherapy practices for chronic spinal pain management. The findings contribute to a framework that can inform future guidelines and orient research, education, and policy toward more sustainable care.

**Significance Statement:**

Chronic spinal pain management is increasingly promoted within conservative care models, yet its environmental sustainability remains largely unexplored in pain research. This international modified Delphi study establishes expert consensus on integrating sustainability considerations into physiotherapy for chronic spinal pain. By providing a structured framework, the findings inform future research agendas, guideline development, and professional education, contributing to more sustainable and responsible pain management aligned with planetary health challenges.

## 1 Introduction

Chronic pain, defined as pain persisting or recurring for more than 3 months (Treede et al. 2019), affects about 20% of the people globally (Rometsch et al. 2025; Sluka 2016) and is recognized in ICD‐11 as both a disease and a symptom, with major impacts on physical, psychological, and social well‐being, as well as healthcare systems (Cohen et al. 2021). Spinal pain is among the most common chronic pain conditions treated in physiotherapy and remains a leading global cause of disability (Chen et al. 2022; GBD 2021 Low Back Pain Collaborators 2023). Chronic spinal pain was selected as a feasible starting point because of its high burden, available guidelines, and reliance on common physiotherapy care pathways such as education, exercise, and self‐management.

Physiotherapy, emphasizing movement and physical function, is central to chronic spinal pain management and recommended in clinical guidelines (Lim et al. 2025; Vaegter et al. 2024). Physiotherapists also promote physical activity, sometimes linked to active transport, which may help reduce pain sensitivity (Toner et al. 2021). Nature‐based strategies, including outdoor exposure and horticultural therapy, improve pain, function, and mental health in structured pain programs (Stanhope et al. 2020; Struthers et al. 2024; Verra et al. 2012).

Despite its non‐pharmacological and non‐invasive nature, physiotherapy contributes to greenhouse gas (GHG) emissions through facility energy use, transport, and equipment (Karliner et al. 2019; Maric and Nicholls 2019; Toner et al. 2021). In healthcare, pharmaceuticals, medical equipment, and materials are major sources of emissions, highlighting the relevance of low‐resource care pathways where clinically appropriate (Mortimer et al. 2018). As global healthcare sectors are major contributors to GHG emissions (Karliner et al. 2019; Romanello et al. 2024), incorporating sustainability into physiotherapy practices can help mitigate these impacts, especially for high‐burden conditions like chronic spinal pain.

Health professionals, including physiotherapists, can help keep human activity within planetary boundaries but need support to promote behavioural and systemic change (Chi et al. 2024; Kotcher et al. 2021; Lee et al. 2021; Palstam and Lange 2024). In physiotherapy, this includes encouraging environmentally sustainable practices and working toward a carbon‐neutral profession (Foo 2016; Jones 2009; Palstam et al. 2022). With the increasing prevalence of spinal pain, addressing the environmental impact of its management strategies is warranted to align healthcare practices with sustainability goals (Romanello et al. 2024; Stanhope et al. 2020).

Despite growing awareness, how sustainability is integrated into physiotherapy for chronic spinal pain remains unclear. This gap extends across research, clinical practice, and education, underscoring the need for a comprehensive understanding of physiotherapists' attitudes and beliefs regarding these issues. Previous work has suggested that physiotherapy has the potential to adopt more sustainable practices by minimizing energy consumption, reducing waste, and leveraging digital health solutions (Maric and Nicholls 2019; Palstam et al. 2022; Toner et al. 2021); however, the extent of integration into spinal pain management remains underexplored.

Gaining insights into physiotherapists' views on sustainability would help develop actionable strategies to reduce the environmental footprint of care, advance research efforts, and enrich educational curricula, all while maintaining high‐quality patient outcomes. This study seeks consensus among physiotherapists on integrating sustainability into chronic spinal pain management to inform guidelines and policies aligned with planetary health goals.

## 2 Methodology

### 2.1 Study Design

A modified Delphi methodology was used to gain a consensus among physiotherapists regarding the integration of environmental considerations in managing chronic spinal pain conditions. Such an approach is justified by the current lack of empirical evidence on how environmental sustainability should be integrated into physiotherapy, particularly in the context of chronic spinal pain. This knowledge gap creates uncertainty across clinical practice, research, and education, making it difficult to identify and prioritize strategic directions. The Delphi method is appropriate in this context, as it enables the gradual convergence of perspectives through iterative rounds, especially in emerging or underexplored fields (Keeney et al. 2011). This iterative approach also supports the progressive refinement of statements and enhances the transparency of the consensus‐building process. The design and reporting of this modified Delphi study were guided by the ACcurate COnsensus Reporting Document (ACCORD), a consensus reporting guideline (Gattrell et al. 2024).

### 2.2 Developing Framework With Explorative Literature Review

A two‐step literature review was conducted to collect evidence for the development of Delphi consensus statements.

#### 2.2.1 Identification of Clinical Guidelines for Chronic Pain Management

A systematic review of clinical practice guidelines (CPGs) for the non‐pharmacological conservative management of adults with chronic spinal pain was conducted. Multiple electronic databases (MEDLINE, EMBASE, PEDro, CINAHL, APA PsycINFO, Epistemonikos) and relevant guideline repositories and websites were systematically searched from January 2018 to November 2023 (Search terms are provided in Supporting Information, Method S1). Eligible documents were evidence‐based CPGs addressing the assessment, evaluation, prevention, or conservative non‐pharmacological management of chronic spinal pain in adults aged 18 years or older. Guidelines focusing primarily on medication alone, interventional procedures, or surgery alone were excluded, as were consensus statements, position statements, and practice alerts. Full eligibility criteria, including pain conditions excluded from the review, are provided in Supporting Information, Method S1.

Screening was performed in three stages: (1) title and abstract; (2) full text; and (3) extraction of relevant CPG recommendations. Two independent reviewers conducted the screening, with a third reviewer resolving disagreements. Eligible CPGs were critically appraised using the Appraisal of Guidelines Research and Evaluation II (AGREE II) (Brouwers et al. 2010). Recommendations from high‐quality evidence‐based CPGs were extracted and synthesised into matrices to facilitate comparison.

#### 2.2.2 Identification of Environmental Impacts of Physiotherapy Treatments

A preliminary literature search was conducted to evaluate the necessity of a systematic review on the environmental impacts of physiotherapy treatments. The search strategy encompassed topics such as waste management, carbon footprint, and resource conservation. This search was performed in Ovid MEDLINE (search date: 27 February 2024; search terms provided in Supporting Information, Methods S1) and identified 264 articles. All titles and abstracts were screened, but no empirical studies directly measuring the environmental impacts of physiotherapy treatments were found. Later, a scoping review by Li et al. (2024) (Li et al. 2024) was published, containing the existing literature between physiotherapy and planetary health (‘the health of human civilization and the state of the natural systems on which it depends’ (Horton et al. 2014; Whitmee et al. 2015)). Most publications in this area consisted of commentaries. Eleven articles either promoting sustainable use of natural resources in clinical practice or presenting interventions of environmental benefit and planetary health were reviewed in detail to inform the foundation of the statements presented in this study.

#### 2.2.3 Delphi Consensus Statement Development

Findings from both reviews informed the development of Delphi consensus statements. Two research team members (EO & AP) integrated this information with their clinical and teaching experience to formulate 47 statements, six open questions, and one ordering question in American English for the initial Delphi questionnaire (see Supporting Information, Methods S2). For example, the following excerpt: ‘(…) we should consider the environmental impact of our practice in our decision‐making and create processes that minimize environmental harm. Clinicians need evidence‐based guidelines to do this (…)’ (Jones 2009) was used to generate the following statement for our e‐Delphi study: ‘Clinical practice guidelines for the management of chronic spinal pain should undergo regular review to ensure alignment with the latest environmental standards and considerations in healthcare research.’

The statements were framed in assertive form (e.g., ‘Physiotherapists/Physical therapists should regularly consult and integrate clinical practice guidelines for chronic spinal pain that incorporate environmental impact considerations into all aspects of patient care.’) to facilitate clear evaluation by participants. These statements were subsequently reviewed and commented on by two bilingual physiotherapists who are native English speakers and fluent in French. The initial statements were then revised and corrected based on their feedback. The revised questionnaire was later tested by three additional physiotherapists to estimate the time required for completion and to ensure clarity and comprehension of the statements. None of these physiotherapists, nor the research team members, participated in the Delphi process to avoid potential bias. After testing the first round in a pilot, participants were recruited. Three successive rounds were required for this study.

### 2.3 Recruitment

There is no universal consensus on the ideal panel size for Delphi studies (Gattrell et al. 2024; Keeney et al. 2011). Although this project was based in Switzerland, our modified electronic approach enabled us to reach international participants. Particular attention was paid to fostering heterogeneity within the panel, by including physiotherapists from different working contexts (e.g., clinical practice, education, and research) and from diverse geographical regions. This strategy was designed to broaden perspectives and enhance the generalizability of the consensus achieved, thereby contributing to the study's external validity (Beiderbeck et al. 2021). Given our aim to capture perspectives from practitioners, educators, and researchers, we targeted a panel size of 45 participants. This number was determined based on the recommendation of including a minimum of 15 to 20 participants per designated subgroup (Beiderbeck et al. 2021).

The eligibility criteria were designed to identify individuals with relevant expertise in management, education, or research of spinal pain. Participants were eligible if they met at least one of the following criteria: (i) a minimum of three years of clinical experience managing chronic spinal pain conditions, including pain related to the cervical spine (neck), thoracic spine (upper‐back), lumbar spine (lower back), or sacral/pelvic region; (ii) at least three years of experience in teaching or educating on pain‐related topics; (iii) authorship of or involvement in at least three peer‐reviewed publications within the field of spinal pain, regardless of authorship position; or (iv) holding the position of head of a musculoskeletal or relevant physiotherapy department. Panellist selection and eligibility screening were conducted by two researchers (EO and AP), in accordance with the predefined inclusion criteria.

Initially, we compiled a list of 124 potential contributors through a systematic search of professional networks, academic publications, and institutional affiliations related to spinal pain research, teaching, and practice. To ensure global representation, we invited panellists to suggest additional contributors. Furthermore, we asked associations (e.g., the Environmental Physiotherapy Association, EcoKiné, the Allied Health Professions Sustainability Network, the European Network of Physiotherapy in Higher Education, and the Physiotherapy Pain Association) to inform their members about the ongoing Delphi project. In total, 150 physiotherapists were invited to participate in Round 1 of the Delphi process. In subsequent rounds, no further modifications were made to the participant list, except for updates to email addresses to maintain effective communication.

Potential participants were contacted via email and provided with a detailed information letter. To maximize response rates, invitations were reiterated with personalized follow‐up emails to encourage non‐responders to participate. Participants who declined the invitation were not included in subsequent rounds of the Delphi process.

Participation in this study was entirely voluntary, and participants could withdraw at any stage without providing a reason or facing any repercussions. Clear instructions regarding voluntary participation were included in the informed consent process. Participants provided informed consent electronically. Upon confirming their participation via the provided platform link, each participant was automatically assigned a unique identification code.

### 2.4 Data Collection

Three rounds were undertaken, between July 2024 and December 2024. Data were collected electronically using the eDelphi platform (version 1.1, released October 11, 2023, Metodix Ltd. and eDelphi Method Software, Espoo, Finland). Invitations to participants were sent via email and through the eDelphi platform, with the option to accept or decline the invitation directly on the platform. Each participant received an individual link to the survey, where they first completed the informed consent step before proceeding to respond to the first Delphi questionnaire. While the links were individualized for secure access and participation tracking, responses were anonymous such that the authors could not identify participants based on their code.

The Delphi process was iterative and conducted over three rounds, each with a specific objective. Three rounds were chosen to allow initial rating, re‐rating of revised statements, and final confirmation while limiting participant burden and attrition, in line with common Delphi practice (Keeney et al. 2011). In Round 1, the primary objective was to assess both the level of agreement and the perceived importance of each statement. Participants rated each statement on these two dimensions, provided comments, and suggested revisions or new ideas. Statements that reached consensus in Round 1 were omitted, while new or revised statements were included in Round 2. The objective of Round 2 was to allow participants to re‐evaluate modified statements that had not reached consensus, with the opportunity to consider the group's feedback (see example in Supporting Information, Methods S3) and to provide arguments for and against their positions. Additionally, newly generated statements or questions were rated. Finally, Round 3 aimed to determine participants' final positions on the remaining statements after reviewing the arguments gathered in previous rounds.

In each round, participants evaluated different types of items. The primary format consisted of statements rated on two distinct 5‐point Likert scales. The first scale assessed the level of agreement with each statement, ranging from ‘strongly disagree’ to ‘strongly agree’. The second scale evaluated the importance of each statement, ranging from ‘not at all important’ to ‘extremely important’. Each statement also included a comment box for feedback and suggestions. Another type of item required participants to prioritize assertions related to a specific theme. Additionally, open‐ended items allowed participants to propose new statements or suggestions on designated topics. A limited number of binary Yes/No questions were also incorporated at specific stages of the Delphi process to refine consensus.

As part of the iterative process, at the beginning of Rounds 2 and 3, participants received a report of the group's overall quantitative responses, including descriptive statistics reflecting the collective opinion and categorized group comments on non‐consensual and modified items.

### 2.5 Data Analysis

It is recommended to establish a clear definition of consensus before commencing the Delphi process (Diamond et al. 2014). Our Delphi study was designed to facilitate consensus among participants; however, we also recognized that the absence of consensus could provide valuable insights into debatable topics. Therefore, we set a limit of three rounds for the process. While consensus thresholds vary in the literature (Keeney et al. 2011), we applied different criteria depending on the type of question.

For this study, data collected from Likert‐scale questions, which constituted the primary method of evaluation, were analysed to determine consensus on each statement. As described above, each statement was rated on two distinct 5‐point Likert scales assessing level of agreement and degree of importance. For each statement and for each dimension, the first quartile (Q1) and third quartile (Q3) were calculated from participant responses, using the default quantile method in R Statistical Software and the interquartile range was computed using the base IQR function as IQR = Q3 − Q1. Consensus was considered achieved only when the IQR was ≤ 1 for both dimensions simultaneously, namely level of agreement and degree of importance. To be classified as a consensus recommendation, a statement also had to have a median score above 3 on both dimensions. This threshold balances the need for a high level of agreement while also allowing for a diversity of opinions among participants. Statements meeting both the IQR and median criteria were considered stable and were removed from subsequent rounds.

For statements where consensus was not reached according to these criteria, they were reviewed by two authors (EO and AP) alongside participant comments. If panellists requested clarifications or suggested specific improvements, and if consensus had not been reached, the statements were revised and reintroduced in the next round.

Statements with a median score of 3, corresponding to ‘neither agree nor disagree’ or ‘I don't know’, were not considered consensual recommendations, even when the IQR was ≤ 1. These items were interpreted as reflecting uncertainty rather than positive consensus. They were therefore classified separately as uncertain items and were excluded from the final count of consensus recommendations.

Answers to open questions were reviewed carefully. In rounds with numerous suggestions for new statements, the comments were categorized into topics for voting. Participants were informed in advance that ‘Topics receiving more than 70% of favourable votes would be transformed into new statements for the final round’.

For multiple‐selection questions, the percentage of votes for each option was considered, with the consensus threshold set at 70%.

For prioritization questions (where participants ranked a series of elements), a point‐based ranking system was employed. Participants were asked to rank the elements based on priority, and points were assigned accordingly. For instance, if a panellist ranked the elements as 3, 1, 4, 2, 5, 6, the highest‐ranked element (element 3) received 6 points, the second (element 1) received 5 points, the third (element 4) received 4 points, and so on. The final score for each element was the sum of all points assigned by participants. This method ensured a quantitative assessment of prioritization, reflecting the relative importance assigned by the panellists.

To monitor trends in agreement and support item prioritization, all survey data from each round were descriptively analysed.

After each round, the survey data were statistically analysed. The statistical group responses were summarised numerically and graphically using measures of central tendency (median, mean), dispersion (interquartile range, standard deviation) and frequency distribution (histograms and bubble charts).

### 2.6 Ethics

The study was reviewed for funding by experts and the scientific committee of the University of Applied Sciences and Arts Western Switzerland. Formal research ethics approval was not required for this study, as it involved a survey of healthcare professionals recruited through established networks. This aligns with the Swiss ethical guidelines for human research, which do not require formal review for professional opinion‐based Delphi studies without personal health data. Participation was voluntary, and responses were collected anonymously.

## 3 Results

### 3.1 Explorative Review of the Literature

#### 3.1.1 Identification of Clinical Guidelines for Chronic Pain Management

Out of the 1090 records initially screened, 14 CPGs met the inclusion criteria and were critically appraised using the AGREE II tool (American College of Occupational and Environmental Medicine 2019; Bundesministerium für Arbeit Soziales Gesundheit und Konsumentenschutz 2018; Bussieres et al. 2018; Colorado Division of Workers' Compensation 2021a, 2021b; Colvin et al. 2019; Department of Veterans Affairs/Department of Defense 2022; George et al. 2021; Korownyk et al. 2022; National Institute for Health and Care Excellence 2020, 2021; North American Spine Society 2020; The Japan Society of Acupuncture and Moxibustion et al. 2021; World Health Organization 2023b). Two CPGs exhibited moderate quality (Bundesministerium für Arbeit Soziales Gesundheit und Konsumentenschutz 2018; The Japan Society of Acupuncture and Moxibustion et al. 2021). On average, the domain ‘Scope and Purpose’ received the highest AGREE II ratings, while ‘Applicability’ was the lowest rated. Among 12 high‐quality CPGs (including two focused on neck pain (Colorado Division of Workers' Compensation 2021a; National Institute for Health and Care Excellence 2021), nine on low back pain (American College of Occupational and Environmental Medicine 2019; Bussieres et al. 2018; Colorado Division of Workers' Compensation 2021b; Department of Veterans Affairs/Department of Defense 2022; George et al. 2021; Korownyk et al. 2022; National Institute for Health and Care Excellence 2020; North American Spine Society 2020; World Health Organization 2023b), and one covering both neck and low back (Colvin et al. 2019)), a total of 201 relevant non‐pharmacological conservative recommendations were extracted. These recommendations were grouped into the following categories: Education and advice (e.g., pain neuroscience education), Physical activity (e.g., structured exercise programs, motor control exercises), Physical agents and modalities (e.g., heat therapy, electrical stimulation, taping), Manual interventions (e.g., joint mobilization, spinal manipulation, massage), Psychological interventions (e.g., cognitive behavioural therapy), Alternative and complementary methods (e.g., acupuncture, reflexology, mindfulness), and Multicomponent interventions (e.g., interdisciplinary programs, multimodal therapy combining physical and psychological approaches).

Manual therapy was recommended in 12 high‐quality CPGs, except for manual traction. Physical activity was endorsed by 11 CPGs, though the type of activity varied. Psychological interventions were supported by 8 CPGs, and education and advice were recommended by 9. Physical agents and modalities were discussed in 10 CPGs, but with some conflicting positions. Alternative and complementary methods were addressed in 8 CPGs, also with divergent views. Multimodal intervention was recommended in 6 CPGs. A summary of these recommendations and AGREE II ratings is available in Supporting Information (Results S1). These findings offered a structured foundation for identifying practice elements relevant to the non‐pharmacological management of chronic spinal pain and informed the development of the initial Delphi statements.

While some clinical guidelines address economic costs, none incorporated environmental impacts. This gap supported the need for a complementary literature review to inform the Delphi process.

#### 3.1.2 Identification of Environmental Impacts of Physiotherapy Treatments

Building on this gap, and in line with methodological recommendations for consensus studies (Beiderbeck et al. 2021; Gattrell et al. 2024), a preliminary literature search was conducted to assess the availability of empirical data on the environmental impacts of physiotherapy treatments. Among the 264 articles identified through Ovid MEDLINE (search date: 27 February 2024), no studies were found that directly measured the environmental footprint of physiotherapy interventions. This confirmed the need to integrate complementary sources to inform the Delphi process.

Following this literature search, the publication of a scoping review (Li et al. 2024) helped consolidate existing knowledge on the intersection between physiotherapy and planetary health. Most of the literature consisted of narrative commentaries or position statements. Eleven articles were examined in depth, either promoting environmentally responsible physiotherapy practices or describing interventions aligned with planetary health principles.

This gap further supported the need for a consensus‐building process. Taken together, both reviews confirmed the lack of empirical environmental data, highlighted emerging advocacy for sustainable physiotherapy, and provided theoretical and practical insights to inform the initial formulation of Delphi statements across clinical practice, research, and education.

### 3.2 Modified Delphi

The Delphi statements were developed based on two complementary sources: (1) key clinical elements identified in high‐quality CPGs on chronic spinal pain, and (2) sustainability principles identified in the retrieved scoping review (Li et al. 2024). These inputs were synthesized into an initial set of 47 statements, along with six open‐ended questions and one prioritization task (see Supporting Information, Methods S2). All items were organized across three dimensions (clinical practice, research, and education) and grouped into six thematic categories: (A) Environmental advocacy and policy, (B) Education and professional development, (C) Research and innovation in treatment methods, (D) Community engagement and patient empowerment, (E) Sustainable clinical practices, and (F) Priorities.

#### 3.2.1 Participants' Characteristics

Of the 150 physiotherapists invited, 44 (29%) agreed to participate in Round 1. Over the course of the three rounds, a total of 49 physiotherapists contributed to at least one round of the Delphi process. Table 1 presents the background characteristics of participants, based on responses to the demographic and professional profile questionnaire.

**TABLE 1:** Participant characteristics of the Delphi panel.

	Round 1	Round 2	Round 3	Global participation
Self‐identified description, *n* (%)				
Female	20 (46%)	13 (37%)	11 (37%)	21 (43%)
Male	23 (52%)	18 (51%)	16 (53%)	24 (49%)
Prefer not to answer	0 (0%)	0 (0%)	0 (0%)	0 (0%)
Unknown	1 (2%)	4 (11%)	3 (10%)	4 (8%)
Age (in years)				
Mean	45.61	45.93	46.39	46.05
SD	10.43	10.94	11.67	10.63
Min	29	29	29	29
Max	73	73	73	73
Missing	8	8	7	12
Years since Bachelor (or equivalent) qualification				
Mean	21.53	22.41	23.04	22.18
SD	10.41	11.61	11.66	11.06
Min	3	3	3	3
Max	46	47	47	47
Missing	6	8	6	10
Primary professional role, *n* (%)				
Clinician	19 (43%)	15 (43%)	12 (40%)	19 (39%)
Educator	12 (27%)	8 (23%)	6 (20%)	12 (24%)
Head of department	1 (2%)	1 (3%)	1 (3%)	1 (2%)
Researcher	12 (27%)	8 (23%)	9 (30%)	14 (29%)
Unknown	0 (0%)	3 (9%)	2 (7%)	3 (6%)
Spinal region most frequently treated/worked with, *n* (%)				
Cervical spine or Neck region	4 (9%)	4 (11%)	3 (10%)	4 (8%)
Thoracic spine or Dorsal region	0 (0%)	0 (0%)	0 (0%)	0 (0%)
Lumbar spine or Lower back region	18 (41%)	13 (37%)	13 (43%)	20 (41%)
Sacral or Pelvic region	1 (2%)	0 (0%)	1 (3%)	1 (2%)
Multiple regions	21 (48%)	15 (43%)	11 (37%)	21 (43%)
Unknown	0 (0%)	3 (9%)	2 (7%)	3 (6%)
Primary continent of professional activity, *n* (%)				
Asia	2 (5%)	1 (3%)	2 (7%)	2 (4%)
Africa	1 (2%)	0 (0%)	0 (0%)	1 (2%)
Europe	24 (55%)	19 (54%)	16 (53%)	26 (53%)
North America	4 (9%)	3 (9%)	2 (7%)	4 (8%)
South America	0 (0%)	0 (0%)	0 (0%)	0 (0%)
Australia/Oceania	3 (7%)	3 (9%)	3 (10%)	3 (6%)
Unknown	10 (23%)	9 (26%)	7 (23%)	13 (27%)
Highest level of education, *n* (%)				
Bachelor's degree or equivalent	3 (7%)	3 (9%)	1 (3%)	3 (6%)
Master's degree or equivalent	15 (34%)	11 (31%)	11 (37%)	16 (33%)
PhD student	6 (14%)	4 (11%)	0 (0%)	6 (12%)
PhD	20 (45%)	14 (40%)	16 (53%)	21 (43%)
Unknown	0 (0%)	3 (9%)	2 (7%)	3 (6%)
Level of interest in environmental sustainability and/or ecology, *n* (%)				
Strongly disinterested	0 (0%)	0 (0%)	0 (0%)	0 (0%)
Disinterested	0 (0%)	0 (0%)	0 (0%)	0 (0%)
Neutral or indifferent	6 (14%)	4 (11%)	4 (13%)	6 (12%)
Interested	23 (52%)	16 (46%)	14 (47%)	24 (49%)
Strongly interested	15 (34%)	12 (34%)	10 (33%)	16 (33%)
Unknown	0 (0%)	3 (9%)	2 (7%)	3 (6%)

Abbreviations: Max, maximum; Min, minimum; SD, standard deviation.

The gender distribution was nearly equal: 43% female, 49% male, and 8% not specified. The mean age was 46.1 years (SD = 10.6, range = 29–73), based on 37 responses. On average, participants obtained their initial qualification 22.2 years earlier (SD = 11.1; range: 3–47), with 10 missing responses.

In terms of professional roles, 39% identified as clinicians, 29% as researchers, 24% as educators, and 2% as heads of departments; 6% did not specify their role. Regarding educational backgrounds, 43% held a PhD, 33% held a master's degree, and 6% held a bachelor's degree; 6% were unspecified.

Many participants reported working across multiple spinal regions (43%), followed by the lower back (41%), and the neck (8%). No participants indicated working primarily with the thoracic region.

Geographically, most participants were based in Europe (53%), followed by North America (8%), Australia/Oceania (6%), Asia (4%), and Africa (2%). Thirteen participants (27%) did not specify their country of practice.

Interest in environmental sustainability and ecology was high: 49% indicated they were ‘interested’ and 33% were ‘strongly interested’. No participants reported being disinterested or strongly disinterested.

#### 3.2.2 Delphi Round Participation and Flow

**FIGURE 1:**
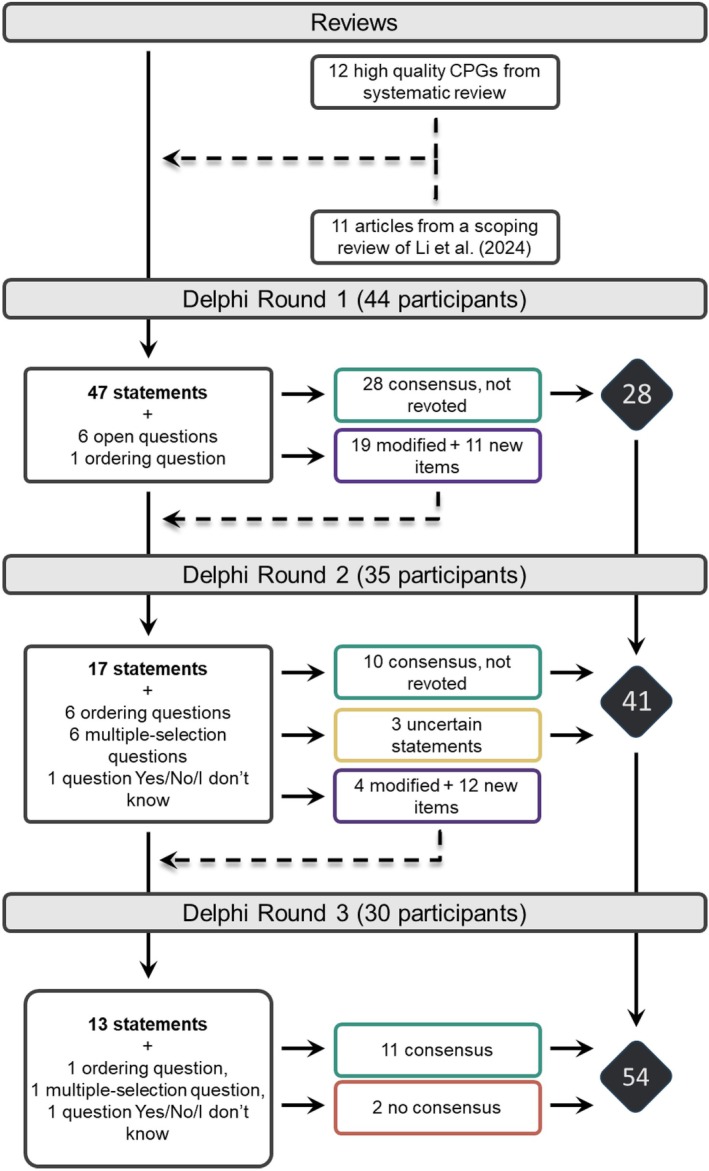
Overview of the Delphi study process and outcomes across three rounds. Initial items were generated from a review of high‐quality clinical practice guidelines and a scoping review of the literature. Round 1 involved 44 participants and included statements, open questions, and ordering questions, leading to initial consensus items and the generation of modified and new items. Round 2 (35 participants) further refined the items, resulting in additional consensus, uncertain statements, and new or modified items. Round 3 (30 participants) led to final consensus on the majority of remaining items. The cumulative number of consensus statements is indicated for each round.

The Delphi process was conducted in three rounds, with a progressive reduction in the number of items and participants across rounds (Figure 1—flow chart, Table 2). Participation rates and the number of statements evaluated in each round are summarized below.

**TABLE 2:** Descriptive statistics of agreement and importance ratings for final statements across Delphi rounds.

Nb	Domain	Statement	Round	Level of agreement	Degree of importance
1	D	Physical therapists, researchers, and interprofessional teams should develop care plans in partnership with patients, focusing on self‐efficacy and self‐management to empower individuals with chronic spinal pain.	R1	Strongly agree Median = 5, IQR = 0 Mean = 4.74, SD = 0.50	Extremely important Median = 5, IQR = 0 Mean = 4.74, SD = 0.50
2	E	Physical therapists should actively minimize overuse and unnecessary procedures in the management of chronic spinal pain (e.g., considering the principles of initiatives like ‘Smarter Medicine’ or ‘Choosing Wisely’).	R1	Strongly agree Median = 5, IQR = 0.5 Mean = 4.69, SD = 0.57	Extremely important Median = 5, IQR = 1 Mean = 4.67, SD = 0.58
3	C	Physical therapists and researchers should develop comprehensive prevention strategies that address the interplay of biological, psychological, social, and environmental factors contributing to chronic spinal pain to reduce its occurrence and improve patient outcomes.	R1	Strongly agree Median = 5, IQR = 1 Mean = 4.63, SD = 0.54	Extremely important Median = 5, IQR = 1 Mean = 4.55, SD = 0.55
4	D	Physical therapy should be promoted as a sustainable treatment solution for chronic spinal pain by reducing reliance on imaging and medication.	R1	Strongly agree Median = 5, IQR = 1 Mean = 4.59, SD = 0.64	Extremely important Median = 5, IQR = 1 Mean = 4.49, SD = 0.68
5	A	Physical therapy associations and relevant stakeholders should develop adaptable and flexible guidelines for sustainable physical therapy practices, ensuring they are feasible and effective across diverse socioeconomic conditions and healthcare systems.	R3	Strongly agree Median = 5, IQR = 1 Mean = 4.50, SD = 0.73	Important Median = 4, IQR = 1 Mean = 4.30, SD = 0.65
6	D	Physical therapists should educate patients with chronic spinal pain about the health and environmental benefits of active transport (e.g., cycling) and outdoor exposure (e.g., hiking).	R1	Strongly agree Median = 5, IQR = 1 Mean = 4.46, SD = 0.72	Extremely important Median = 5, IQR = 1 Mean = 4.41, SD = 0.75
7	E	Physical therapists involved in chronic spinal pain care should identify and implement basic sustainable practices (e.g., reducing electricity consumption, minimizing waste, etc.).	R1	Strongly agree Median = 5, IQR = 1 Mean = 4.46, SD = 0.72	Important Median = 4, IQR = 1 Mean = 4.28, SD = 0.86
8	B	Physical therapists should be educated about the potential strengths, opportunities, weaknesses and threats of Artificial Intelligence in the management of chronic spinal pain (e.g., pre‐screening CLBP by Autonomous AI may be an opportunity to reduce healthcare carbon footprint).	R2	Agree Median = 4, IQR = 1 Mean = 4.39, SD = 0.56	Important Median = 4, IQR = 1 Mean = 4.29, SD = 0.74
9	E	Technology developers should ensure that digital health solutions for chronic spinal pain management are designed with eco‐friendly features and minimal electronic footprints.	R1	Strongly agree Median = 5, IQR = 1 Mean = 4.38, SD = 0.75	Important Median = 4, IQR = 1 Mean = 4.13, SD = 0.86
10	A	Policymakers and healthcare organizations should develop and implement policies that promote sustainable practices at both the service and individual levels, ensuring alignment with environmental goals and clinical effectiveness.	R3	Agree Median = 4, IQR = 1 Mean = 4.37, SD = 0.56	Important Median = 4, IQR = 1 Mean = 4.07, SD = 0.78
11	A	Public health officials and policymakers should assume the leadership to assess the physical, economic, and environmental benefits of safe active transport infrastructure (e.g., bicycle lanes), in collaboration with multiple professionals (e.g., urban planners, researchers, etc.).	R3	Agree Median = 4, IQR = 1 Mean = 4.34, SD = 0.77	Important Median = 4, IQR = 1 Mean = 4.17, SD = 0.80
12	E	Physical therapists should explore and integrate digital health tools (e.g., remote consultation systems, apps…) in the management of chronic spinal pain conditions to reduce time, financial, and environmental costs, while ensuring such tools also support favourable patient outcomes and maintain clinical effectiveness.	R1	Strongly agree Median = 5, IQR = 1 Mean = 4.33, SD = 0.90	Important Median = 4, IQR = 1 Mean = 4.23, SD = 0.93
13	D	Physical therapists should consider incorporating nature‐based interventions (e.g., exposure to green and blue spaces) for treating patients with chronic spinal pain conditions, provided these approaches are evidence‐based and aligned with individual patient preferences.	R2	Agree Median = 4, IQR = 1 Mean = 4.32, SD = 0.60	Important Median = 4, IQR = 1 Mean = 4.06, SD = 0.85
14	E	Physical therapists involved in chronic spinal pain care should promote and use active transport, such as cycling or walking, whenever possible, thereby offering co‐benefits of environmental sustainability and improved personal well‐being.	R1	Agree Median = 4, IQR = 1 Mean = 4.31, SD = 0.77	Important Median = 4, IQR = 1 Mean = 4.23, SD = 0.71
15	A	Policymakers (e.g., governments and regulatory bodies) should have the leading responsibility for pressuring suppliers to minimize packaging and adopt energy‐efficient equipment.	R3	Agree Median = 4.5, IQR = 1 Mean = 4.30, SD = 0.84	Important Median = 4, IQR = 1 Mean = 4.20, SD = 0.81
16	B	Physical therapists should educate themselves and their healthcare colleagues to effectively integrate sustainable practices in managing chronic spinal pain.	R1	Agree Median = 4, IQR = 1 Mean = 4.28, SD = 0.72	Important Median = 4, IQR = 1 Mean = 4.15, SD = 0.81
17	D	Physical therapists involved in chronic spinal pain care should play a role in community projects that encourage physical activity and ecological restoration (e.g., public health strategies, urban planning, etc.).	R1	Agree Median = 4, IQR = 1 Mean = 4.28, SD = 0.72	Important Median = 4, IQR = 1 Mean = 4.15, SD = 0.71
18	C	Researchers should explore the effectiveness, barriers, and enablers of nature‐based interventions (e.g., outdoor exposure) in managing chronic spinal pain, to optimize their practical application and outcomes.	R1	Agree Median = 4, IQR = 1 Mean = 4.26, SD = 0.64	Important Median = 4, IQR = 1 Mean = 4.13, SD = 0.74
19	B	Physical therapy training should equip practitioners with the skills to utilize outdoor exercises and natural environments, whenever possible and safe, as effective treatment strategies for chronic spinal pain.	R2	Agree Median = 4, IQR = 1 Mean = 4.26, SD = 0.63	Important Median = 4, IQR = 1 Mean = 4.00, SD = 0.86
20	A	Physical therapy organizations should create alliances and formulate policies with stakeholders at multiple levels (global, national, and local) to sustainably address chronic spinal pain.	R1	Agree Median = 4, IQR = 1 Mean = 4.25, SD = 0.89	Important Median = 4, IQR = 1 Mean = 4.11, SD = 0.78
21	B	Continuing education courses should provide training in sustainable approaches for managing chronic spinal pain to ensure that physical therapists remain up to date on these evolving practices after graduation.	R1	Agree Median = 4, IQR = 1 Mean = 4.23, SD = 0.67	Important Median = 4, IQR = 1 Mean = 3.87, SD = 0.86
22	B	Physical therapy education programs should integrate content on environmental health into the management of chronic musculoskeletal pain conditions.	R2	Agree Median = 4, IQR = 1 Mean = 4.22, SD = 0.83	Important Median = 4, IQR = 1 Mean = 3.75, SD = 1.02
23	A	Physical therapists should actively incorporate the Sustainable Development Goals into their research to enhance the environmental and societal impact of physical therapy for chronic spinal pain management.	R1	Agree Median = 4, IQR = 1 Mean = 4.19, SD = 0.59	Important Median = 4, IQR = 0 Mean = 3.98, SD = 0.75
24	B	Clinical practice guidelines for the management of chronic spinal pain should undergo regular review to ensure alignment with the latest environmental standards and considerations in healthcare research.	R1	Agree Median = 4, IQR = 1 Mean = 4.18, SD = 0.76	Important Median = 4, IQR = 0.5 Mean = 3.92, SD = 0.9
25	C	Researchers in chronic spinal pain care should conduct studies to assess whether the digitalization of physical therapy services offers a net positive impact on environmental sustainability.	R1	Agree Median = 4, IQR = 1 Mean = 4.18, SD = 0.61	Important Median = 4, IQR = 0.75 Mean = 3.84, SD = 0.89
26	D	Patient advocacy groups should collaborate with physical therapists and researchers to identify barriers and enablers to implementing environmentally sustainable actions for managing chronic spinal pain.	R1	Agree Median = 4, IQR = 1 Mean = 4.18, SD = 0.64	Important Median = 4, IQR = 0.5 Mean = 4.05, SD = 0.76
27	B	In the absence of payment models for remote consultations (e.g., standard invoice prices), such models should be developed to ensure the financial stability of physical therapists involved in chronic spinal pain care.	R2	Agree Median = 4, IQR = 1 Mean = 4.16, SD = 0.90	Important Median = 4, IQR = 1 Mean = 3.87, SD = 0.85
28	A	Healthcare administrators should adopt policies that incentivize environmentally friendly physical therapy practices for chronic spinal pain management.	R2	Agree Median = 4, IQR = 1 Mean = 4.15, SD = 0.73	Important Median = 4, IQR = 0 Mean = 3.93, SD = 0.85
29	B	Although physical therapy is often considered eco‐friendly (using modalities such as movement, communication, and touch), physical therapists managing chronic musculoskeletal pain conditions should further educate themselves about sustainable practices.	R2	Agree Median = 4, IQR = 1 Mean = 4.13, SD = 0.72	Important Median = 4, IQR = 0 Mean = 3.97, SD = 0.75
30	C	Physical therapists should advocate for increased funding and investment in the development, implementation, and evaluation of eco‐friendly therapies that align with evidence‐based practices.	R3	Agree Median = 4, IQR = 0 Mean = 4.13, SD = 0.57	Important Median = 4, IQR = 0 Mean = 3.90, SD = 0.71
31	B	Healthcare professionals should regularly consult and integrate clinical practice guidelines for chronic spinal pain that incorporate environmental impact considerations into all aspects of patient care, when available.	R2	Agree Median = 4, IQR = 1 Mean = 4.12, SD = 0.71	Important Median = 4, IQR = 1 Mean = 3.75, SD = 1.02
32	C	Supported by academic institutions and healthcare organizations, physical therapists should actively participate in empirical research studies to understand the environmental effects of treatments for chronic spinal pain.	R1	Agree Median = 4, IQR = 0.5 Mean = 4.10, SD = 0.72	Important Median = 4, IQR = 1 Mean = 3.74, SD = 0.85
33	D	Physical therapists should optimize the allocation of healthcare resources by expanding the scope of individual treatments to include community education initiatives, with a focus on reaching and supporting underprivileged populations.	R3	Agree Median = 4, IQR = 0 Mean = 4.10, SD = 0.66	Important Median = 4, IQR = 0.75 Mean = 3.97, SD = 0.85
34	C	Healthcare providers and relevant stakeholders (e.g., sustainability experts) should collaborate to comprehensively evaluate the environmental impact of chronic musculoskeletal pain, i.e., its environmental burden.	R3	Agree Median = 4, IQR = 0.75 Mean = 4.07, SD = 0.74	Important Median = 4, IQR = 0 Mean = 3.80, SD = 0.96
35	E	Healthcare providers, institutions, and relevant stakeholders (e.g., sustainability experts) should develop and implement evidence‐based and feasible strategies to integrate sustainable clinical practices for managing chronic spinal pain into routine workflows, ensuring alignment with environmental objectives while minimizing disruption to care delivery.	R3	Agree Median = 4, IQR = 1 Mean = 4.07, SD = 0.87	Important Median = 4, IQR = 1 Mean = 4.00, SD = 1.02
36	C	Researchers should conduct studies to evaluate the environmental impact and clinical efficacy of telehealth and digitalization in the management of chronic spinal pain, ensuring that both sustainability and treatment effectiveness are prioritized.	R1	Agree Median = 4, IQR = 0.75 Mean = 4.05, SD = 0.70	Important Median = 4, IQR = 0 Mean = 4.00, SD = 0.74
37	D	Physical therapists should develop a comprehensive understanding of global environmental issues and their potential links with chronic diseases (e.g., air pollution and chronic pain), enabling them to collaboratively and appropriately tailor treatment decisions with their patients.	R2	Agree Median = 4, IQR = 0.75 Mean = 4.03, SD = 0.76	Important Median = 4, IQR = 0 Mean = 3.93, SD = 0.78
38	A	Government health departments or other regulatory health bodies should consider developing flexible and evidence‐based national guidelines for healthcare professionals and interprofessional teams to foster environmentally sustainable approaches in the management of chronic musculoskeletal pain.	R3	Agree Median = 4, IQR = 0 Mean = 4.03, SD = 0.72	Important Median = 4, IQR = 0.75 Mean = 4.13, SD = 0.68
39	B	Physical therapists should incorporate environmental considerations into their clinical reasoning when managing chronic spinal pain.	R1	Agree Median = 4, IQR = 0 Mean = 4.00, SD = 0.76	Important Median = 4, IQR = 1 Mean = 3.62, SD = 0.94
40	C	Researchers should measure and compare the environmental impacts of physical therapy treatments with those of other healthcare interventions for chronic spinal pain.	R1	Agree Median = 4, IQR = 0 Mean = 4.00, SD = 0.70	Important Median = 4, IQR = 0 Mean = 3.87, SD = 0.74
41	D	Physical therapists should partner with their patients with chronic spinal pain to select actions that balance both individual needs and environmental concerns (e.g., sustainable nutrition advice for pain management), according to the specific contexts.	R2	Agree Median = 4, IQR = 0 Mean = 4.00, SD = 0.79	Important Median = 4, IQR = 0.75 Mean = 3.90, SD = 0.84
42	C	Healthcare professionals, in collaboration with researchers from other disciplines (e.g., environmental scientists) could explore the development of develop tools to estimate the environmental footprint of clinical practices in chronic musculoskeletal pain (e.g., footprint calculator similar to the World Wildlife Fund's, but tailored to healthcare).	R3	Agree Median = 4, IQR = 0 Mean = 4.00, SD = 0.64	Important Median = 4, IQR = 1 Mean = 3.77, SD = 0.82
43	D	Physical therapists involved in chronic spinal pain care should lead by example in adopting and promoting a culture of environmental sustainability within their practices and communities, as part of a broader effort to mitigate climate change.	R1	Agree Median = 4, IQR = 0 Mean = 3.95, SD = 0.73	Important Median = 4, IQR = 0.75 Mean = 3.76, SD = 0.88
44	D	Non‐governmental organizations focused on the environment and health should raise awareness about the importance of sustainable physical therapy practices in managing chronic spinal pain.	R1	Agree Median = 4, IQR = 1 Mean = 3.87, SD = 0.73	Important Median = 4, IQR = 1 Mean = 3.82, SD = 0.79
45	E	Physical therapists should adopt the sustainable value equation as a guiding framework for decision‐making in chronic spinal pain care.	R1	Agree Median = 4, IQR = 0 Mean = 3.87, SD = 0.80	Important Median = 4, IQR = 1 Mean = 3.77, SD = 0.93
46	C	Future research should investigate barriers within the physical therapy management of chronic spinal pain with a focus on embedding sustainability into all aspects of treatment protocols and clinical procedures.	R1	Agree Median = 4, IQR = 1 Mean = 3.84, SD = 0.89	Important Median = 4, IQR = 1 Mean = 3.74, SD = 0.89
47	A	Physical therapists could support efforts, in collaboration with policymakers and advocacy groups, to promote transparency about the environmental impact of product manufacturing (e.g., labeling systems to compare the environmental costs).	R3	Agree Median = 4, IQR = 0 Mean = 3.83, SD = 0.91	Important Median = 4, IQR = 1 Mean = 3.37, SD = 1.03
48	A	Research funding agencies should insist on sustainability as a key requirement for granting chronic spinal pain research.	R1	Agree Median = 4, IQR = 1 Mean = 3.79, SD = 0.98	Important Median = 4, IQR = 1 Mean = 3.57, SD = 1.06
49	E	Physical therapists should use Generative Artificial Intelligence (e.g., ChatGPT, Gemini, Copilot…) in the management of chronic spinal pain with caution and consider its environmental impact (e.g., large resource consumption).	R2	Agree Median = 4, IQR = 1 Mean = 3.77, SD = 0.68	Important Median = 4, IQR = 1 Mean = 3.73, SD = 0.74
50	C	Physical therapists should explore Artificial Intelligence (e.g., running trial simulations digitally) to innovate and implement green solutions in the management and research in chronic spinal pain.	R2	Agree Median = 4, IQR = 1 Mean = 3.68, SD = 0.79	I don't know Median = 3, IQR = 1 Mean = 3.42, SD = 0.84
51	D	Physical therapists should investigate the potential of traditional and Indigenous knowledge, where relevant, to enhance holistic management of chronic spinal pain, aligning environment, health, and community factors, while ensuring evidence‐based practice.	R2	Agree Median = 4, IQR = 1 Mean = 3.68, SD = 1.05	I don't know Median = 3, IQR = 1 Mean = 3.89, SD = 1.17
52	C	Journal and review editors should encourage that submitted research on the management of chronic spinal pain demonstrate the consideration of environmentally conscious practices, where relevant.	R2	Agree Median = 4, IQR = 1 Mean = 3.55, SD = 0.93	I don't know Median = 3, IQR = 1 Mean = 3.42, SD = 1.09
53	C	Physical therapists and researchers should consider developing outcome measures to assess the clinical impacts of shared eco‐friendly decision‐making in chronic spinal pain management (e.g., satisfaction, readmission rates, avoided medical or therapeutic procedures).	R3	Agree Median = 4, IQR = 1 Mean = 4.17, SD = 0.87	No consensus Median = 4, IQR = 1.75 Mean = 4.03, SD = 0.85
54	C	Researchers should consider both the therapeutic results and environmental impacts of physical therapy treatments to inform clinical decision‐making in chronic spinal pain management, where relevant, considering long‐term effects, individual patient needs, and the practical challenges of assessing environmental factors.	R3	No consensus Median = 4, IQR = 1.5 Mean = 3.77, SD = 1.10	Important Median = 4, IQR = 1 Mean = 3.83, SD = 0.87

*Note:* Statement status is indicated by grayscale shading: no shading identifies consensus recommendations, light grey identifies uncertain statements, and darker grey identifies statements with no consensus.

Abbreviations: A, environmental advocacy and policy; B, education and professional development; C, research and innovation in treatment methods; D, community engagement and patient empowerment; E, sustainable clinical practices; IQR, interquartile range; SD, standard deviation.

Round 1 involved 44 participants who assessed 47 statements, responded to 6 open‐ended questions, and completed 1 prioritization task. Consensus (defined as IQR ≤ 1 on both agreement and importance) was reached for 28 of the 47 statements (60%).

Round 2 included 35 participants and involved 17 revised statements, 6 prioritization questions, 6 multiple‐choice questions, and 1 Yes/No/I don't know item. Of the 17 statements, 10 (59%) reached consensus, 3 remained uncertain, and 4 required modifications.

Round 3 involved 30 participants and addressed the 13 remaining statements. It also included one prioritization question, one multiple‐choice item, and one Yes/No/I don't know item. By the end of Round 3, consensus had been reached for all but two statements.

Statements reaching consensus were removed from subsequent rounds to avoid fatigue, unless participants requested clarification. Statements without consensus were revised or excluded based on participant feedback and the likelihood of convergence.

The two statements that did not reach consensus at the end of Round 3 are listed below

– Physiotherapists/Physical therapists and researchers should consider developing outcome measures to assess the clinical impacts of shared eco‐friendly decision‐making in chronic spinal pain management (e.g., satisfaction, readmission rates, avoided medical or therapeutic procedures) [No consensus on degree of importance (Median = 4, IQR = 1.75) but consensus on level of agreement ‘Agree’].
– Researchers should consider both the therapeutic results and environmental impacts of physiotherapy/physical therapy treatments to inform clinical decision‐making in chronic spinal pain management, where relevant, considering long‐term effects, individual patient needs, and the practical challenges of assessing environmental factors [No consensus on level of agreement (Median = 4, IQR = 1.5) but consensus on degree of importance with statement considered as ‘Important’].

A summary of the process is provided in Figure 1 (Flow Chart), and detailed consensus levels per item and round (level of agreement and degree of importance) are presented in Table 2.

#### 3.2.3 Thematic Consensus Findings

**FIGURE 2:**
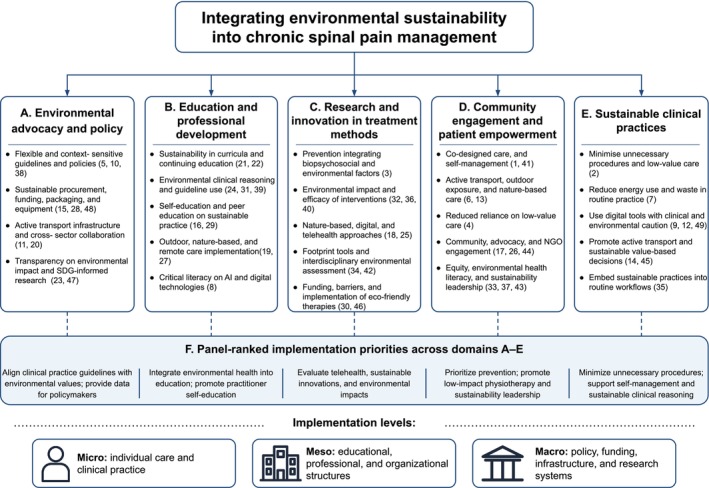
Consensus framework for integrating environmental sustainability into chronic spinal pain management. The figure summarizes the five consensus domains and the panel‐ranked implementation priorities identified through the Delphi process. Domains A to E describe the main areas of action, while Section F summarizes priorities identified across these domains to support implementation across practice, education, research, and policy. The lower part of the figure situates implementation across micro‐level individual care and clinical practice, meso‐level educational, professional, and organizational structures, and macro‐level policy, funding, infrastructure, and research systems. Numbers in parentheses refer to the corresponding consensus statements reported in Table 2.

The Delphi panel reached consensus on 49 statements, which may be thematically grouped into five interrelated domains (A to E) reflecting the multidimensional integration of environmental sustainability into chronic spinal pain care (Figure 2). An additional section outlines priority actions to support implementation across domains.

- Environmental Advocacy and Policy

Ten statements reached consensus under this theme, emphasizing the critical role of macro‐level actors in advancing sustainability within chronic spinal pain care. The panel broadly supported coordinated action by public health authorities, policymakers, healthcare organizations, professional organizations, and research funders to foster environmentally responsible frameworks. Key directions included incentivizing eco‐responsible clinical practices, developing flexible and context‐sensitive national guidelines, promoting active transport infrastructures, and aligning funding and procurement policies with environmental objectives. Cross‐sector collaboration and enhanced transparency regarding the environmental impact of healthcare products (e.g., through labelling systems to compare the environmental costs) were also endorsed, albeit with slightly more varied levels of importance and agreement.

B Education and Professional Development

The panel agreed on 10 statements highlighting the importance of integrating environmental sustainability throughout the continuum of professional training. Sustainability‐related competencies were considered essential at all educational levels, from entry‐level instruction to postgraduate courses and continuing professional development. Supporting Information (Results S2) outlines potential environmental health topics to be integrated into educational programs (see 1.1.1), notably eco‐friendly exercise prescription (e.g., nature‐based exercise), as well as the key environmental considerations that should be addressed in CPGs (see 1.1.3), such as prevention strategies (e.g., education for general public). Participants emphasized the need to prepare physiotherapists to incorporate environmental reasoning into their clinical decisions, to use nature‐based therapies where appropriate, and to develop the critical literacy needed to assess emerging technologies such as artificial intelligence (AI). The panel also identified structural and systemic barriers, including reimbursement models, as key targets for educational reform to ensure that sustainability principles can be applied in real‐world practice.

C Research and Innovation in Treatment Methods

Ten statements reached consensus, highlighting the need to strengthen the evidence on the environmental impact of interventions for chronic spinal pain. A large majority of the panel (83%) agreed that environmental sustainability is an appropriate research focus in physiotherapy, and that sustainability efforts should be considered part of professional responsibilities (see 2.2.1 in Supporting Information, Results S2). The panel emphasized research on prevention strategies integrating biopsychosocial and environmental factors, nature‐based approaches, telehealth, and digital tools, as well as comparative studies with other healthcare modalities. The panel encouraged the development of footprint estimators and called for stronger collaboration across disciplines to assess the broader environmental burden of musculoskeletal pain. While most directions received strong support, items related to AI and sustainability criteria in scientific publishing elicited more varied responses, reflecting areas of emerging interest and debate.

D Community Engagement and Patient Empowerment

Eleven statements reached consensus, highlighting the central role of patients and communities in advancing environmentally sustainable care for chronic spinal pain. The panel strongly supported co‐designed care plans that foster self‐efficacy and patient autonomy, as well as the integration of sustainability‐oriented behaviours such as active transport and reduced reliance on medication and imaging. Participation in ecological and community‐based initiatives was encouraged, along with efforts to raise awareness among underprivileged populations and support equitable resource allocation. Education and leadership by physiotherapists were seen as key to promoting awareness and modelling sustainable practices. A highly debated topic was whether physiotherapists should raise awareness about the environmental impacts of treatments among patients who seem unconcerned about these issues (see 1.3.1 in Supporting Information, Results S2). The panel recommended that discussions on this topic are appropriate when directly related to the patient's health or rehabilitation goals (see 2.2.1). Suggested topics for raising patient awareness about environmental issues included for instance outdoor exposure, benefits of active transport, and psychological well‐being through nature (see 1.1.2 in Supporting Information, Results S2). While most statements were rated as highly important and strongly agreed upon, suggestions involving traditional and Indigenous knowledge received more varied responses, indicating both interest and a need for further empirical grounding.

E Sustainable Clinical Practices

Consensus was reached on eight statements promoting pragmatic strategies to reduce the environmental footprint of chronic spinal pain care. High levels of agreement were observed for minimizing unnecessary procedures, in line with initiatives such as ‘Choosing Wisely,’ and for implementing simple eco‐responsible behaviours (e.g., reducing energy use, minimizing waste). The panel also supported the integration of digital tools, provided their clinical effectiveness and sustainability are both considered. While generative AI was viewed as a potentially useful innovation, participants emphasized the need to remain cautious about its resource intensity. Active transport options and the adoption of frameworks such as the sustainable value equation were also encouraged, although responses to these items showed slightly more variation, suggesting the need for further clarification or support in clinical implementation. Finally, embedding sustainability into clinical workflows was endorsed as a shared responsibility among practitioners, institutions, and system‐level stakeholders.

F Priorities

Finally, six items addressed the priorities in each section A to E (see 1.2.1 to 1.2.6 in Supporting Information, Results S2). In Domain A, the panel identified two top priorities for physiotherapists: aligning clinical practice guidelines with environmental values and providing data‐driven insights to policymakers to support environmentally informed decision‐making. In Domain B, the focus was placed on integrating environmental health into physiotherapy education as a core component of training, along with promoting self‐education and personal responsibility among practitioners. For Domain C, the top priorities included the need for research into telehealth and digital health solutions, as well as exploring innovative and sustainable physiotherapy practices. In Domain D, the panel emphasized the importance of prevention strategies for chronic spinal pain and the promotion of physiotherapy as a sustainable, low‐impact treatment alternative. Lastly, in Domain E, the two main priorities were minimizing unnecessary procedures that may contribute to environmental or healthcare waste and encouraging self‐management while incorporating environmental sustainability into clinical reasoning and decision‐making.

With regard to broader implementation strategies and system‐level priorities, the panel strongly supported developing adaptable, evidence‐based guidelines suitable for diverse healthcare systems and socioeconomic contexts. They emphasized the need for policies that align sustainability goals with clinical effectiveness, as well as targeted investments in eco‐responsible therapies backed by robust evidence. Focus areas also included multisectoral collaboration, equitable resource allocation, and inclusive outreach to underrepresented populations, alongside evaluating the environmental burden of chronic spinal pain and embedding sustainable practices into routine workflows without compromising care quality.

## 4 Discussion and Conclusions

### 4.1 Toward Environmentally Sustainable Chronic Spinal Pain Care

This international modified Delphi identified priority actions for embedding environmental sustainability within chronic spinal pain care. Through a transparent process, the panel reached consensus across five domains—advocacy, education, research, community engagement, and sustainable clinical practices—linking clinical effectiveness and health promotion to sustainability goals. The resulting framework outlines feasible, evidence‐based strategies, cross‐sector collaboration, supportive regulation, and institutional enablers to advance low‐impact, high‐value care.

These directions gain particular relevance considering the healthcare sector's contribution to approximately 5% of global GHG emissions (Karliner et al. 2019; Lenzen et al. 2020) and its growing waste and pollution (Romanello et al. 2024). Yet, our review of CPGs revealed that environmental impacts remain invisible. While some guidelines address economic or social costs, none consider environmental dimensions. A recent scoping review confirmed this omission (Svensson et al. 2024), supporting the gap identified in our preliminary review and the panel's call for methodological innovation.

Bridging this gap requires alignment with global decarbonization strategies such as the *Global Roadmap for Healthcare Decarbonization*, which outlines three interconnected pathways (covering healthcare operations, supply chains, and their wider economic links) to guide the sector toward near‐zero emissions by mid‐century. Achieving this goal requires systemic transformation, innovation, and cross‐sector collaboration while maintaining care quality (Karliner et al. 2021). To translate our findings into action, a systemic interpretation across three levels—micro, meso, and macro—helps clarify where sustainability can be integrated into spinal pain care. This perspective aligns with systemic approaches to chronic pain management that distinguish individual, organizational, and policy determinants of care (Buchbinder et al. 2020; Gatchel et al. 2007), and echoes calls to move beyond individual awareness toward structural and institutional change (Horton et al. 2014; Romanello et al. 2024).

### 4.2 Micro Level: Individual Care and Clinical Practice

At the clinical interface, environmental sustainability begins with everyday clinical reasoning. The panel strongly endorsed practices that combine clinical, environmental, and social values, particularly self‐management, co‐designed care plans that foster equity, participation, and shared decision‐making (Holzer et al. 2025), and the avoidance of low‐value or resource‐intensive procedures such as unnecessary imaging or medication (Foster et al. 2018). These recommendations reflect that sustainability should reinforce, not compete with, principles of safety and quality. They align with ‘low‐impact, high‐value’ approaches in musculoskeletal care (Born and Levinson 2019) and with broader redefinitions of clinical value integrating sustainability and equity (Mortimer et al. 2018).

These principles extend to the content of rehabilitation itself. Panellists particularly valued movement‐ and education‐based interventions as practical expressions of low‐impact, high‐value care. Physical activity, a cornerstone of chronic pain management, represents a low‐risk, low‐cost approach with health and environmental co‐benefits (Toner et al. 2021; Vaegter et al. 2024). When embedded in active transport, it may also contribute to emission reduction. Yet, climate‐related constraints—such as increased exposure to heat stress, with 27.7% more hours of moderate heat stress in 2023 compared with the 1990s baseline—can limit feasibility and exacerbate inequities. Clinicians therefore need to tailor movement‐based recommendations to local environmental conditions and patient capacities to ensure that sustainable choices remain both safe and accessible (Romanello et al. 2024; Toner et al. 2021).

Beyond therapeutic content, technological choices also influence the environmental profile of care. Digital care pathways can reduce travel‐related emissions when clinically appropriate, as shown in musculoskeletal self‐management programs reporting lower carbon footprints while maintaining outcomes (Tucker et al. 2025). Panellists cautioned that digital tools should be used only when clinically and environmentally efficient, with transparent reporting on adherence, literacy, and resource intensity.

### 4.3 Meso Level: Educational, Professional, and Organizational Structures

Panellists emphasized that lasting change depends on meso‐level structures supporting education, research, and coordinated professional action. These institutional enablers determine whether environmental sustainability remains individual or becomes a shared professional standard (Maric et al. 2021; World Health Organization 2023a).

Education and professional development were viewed as central. Panellists supported integrating environmental competencies across education, positioning sustainability as part of professional literacy rather than an elective topic. Evidence identifies similar barriers—limited time and expertise—and facilitators such as accreditation standards, interprofessional learning, and leadership from professional associations (Chi et al. 2024). These directions align with planetary‐health frameworks linking human, social, and ecological well‐being (Guzman et al. 2021).

In research and innovation, participants called for methods to quantify the environmental footprint of interventions and for sustainability criteria in funding and publication. This ‘dual‐value research’ approach combines scientific rigour with ecological accountability (Cooke et al. 2023; Herrmann et al. 2022; Mortimer et al. 2018). Developing these approaches requires cross‐disciplinary collaboration with environmental and engineering sciences, and inclusion of environmental indicators in trials to assess both therapeutic benefit and resource use.

At the organizational level, universities, hospitals, and professional societies were identified as key levers. Governance, procurement policies, and resource management can foster or hinder low‐carbon healthcare environments (World Health Organization 2023a). Within pain care, professional bodies can operationalize sustainability principles by setting standards, fostering collective learning, and guiding institutional transitions toward low‐carbon practice (Romanello et al. 2024).

### 4.4 Macro Level: Policy and System‐Level Strategies

At the macro level, panellists emphasized that sustainable transformation in chronic spinal pain care relies on supportive policy and system environments. They highlighted the role of public health authorities, policymakers, and funders in aligning incentives, regulations, and investment with environmental and social goals, embedding sustainability within national and regional health strategies while ensuring equitable access for underserved populations (Swinburn et al. 2019; Whitmee et al. 2015). They further called for adaptable, context‐sensitive guidelines, cross‐sector collaboration involving the health, transport and education sectors, and greater transparency in procurement and reimbursement systems to foster accountability and incentivize low‐impact clinical options.

These macro‐level directions were mirrored in the panel's prioritization exercise, which identified two overarching goals for health professionals and professional bodies: (1) aligning clinical practice guidelines with environmental values, and (2) providing data‐driven evidence to policymakers to support environmentally informed decision‐making. Panellists stressed equitable access and inclusion of underrepresented groups in implementing sustainability initiatives.

This policy vision is coherent with broader international frameworks such as the UN Agenda 2030 Sustainable Development Goals and the WHO Global Strategy on Health, Environment and Climate Change, which link healthcare decarbonization to population well‐being (United Nations 2015; World Health Organization 2020). Frameworks such as planetary health and One Health reinforce the idea that sustainable pain care must operate within wider social and ecological systems (Maric et al. 2021; Horton et al. 2014).

### 4.5 Environmental Sustainability as an Additional Lens Within Evidence‐Based Practice

The classical evidence‐based practice (EBP) triad—best evidence, clinical expertise, and patient values within a specific context (Sackett et al. 1996)—may be insufficient to guide truly responsible practice when ecological determinants increasingly shape health and care delivery. Our panel proposed conceptualizing environmental sustainability as a fourth, complementary pillar of EBP. This extension invites clinicians and guideline developers to consider, where feasible, the environmental and social footprints, resource efficiency, and contribution to planetary health of care options alongside clinical and economic value, including proposals for embedding environmental indicators and life‐cycle considerations in CPGs (Cooke et al. 2023; Herrmann et al. 2022). These perspectives converge toward an expanded understanding of evidence that integrates environmental and ethical dimensions into decision‐making and supports including sustainability indicators and low‐impact interventions within clinical guidelines (Herrmann et al. 2022).

### 4.6 Interpreting the Two Non‐Consensus Items

Two statements did not reach full consensus. For eco‐informed outcomes, agreement on principle but uncertainty about measurable effects suggests small pilot work on core outcomes such as decision quality and avoidance of low‐value care. For combined clinical, environmental, and social impacts, high importance but limited indicator maturity calls for phased research to test, compare, and refine footprint measures.

### 4.7 Strengths and Limitations

Strengths include a rigorous Delphi process conducted in line with ACCORD guidelines, the synthesis of high‐quality CPGs and a scoping review on planetary health, and a diverse panel spanning clinical, research, and educational roles. The field‐specific focus on chronic spinal pain addresses a high‐burden area of physiotherapy in which sustainability considerations remain insufficiently defined. Organizing results across five domains facilitates practical translation at micro, meso, and macro levels.

Limitations include the predominantly European composition of the panel, attrition across rounds, and probable self‐selection bias among participants already engaged in sustainability. The absence of patient representatives contrasts with the panel's emphasis on empowerment and should be addressed in future research. The literature informing the initial statements, largely in English, may have introduced regional bias. Finally, the lack of empirical environmental data on chronic spinal pain management constrained the formulation of evidence‐based recommendations in some areas, underscoring the need for applied research evaluating both clinical outcomes and environmental impacts (Maric et al. 2025; Svensson et al. 2024). Although developed for chronic spinal pain, some principles may be relevant to other musculoskeletal conditions; however, their transferability should be examined in future studies.

In conclusion, this study highlights the interdependent dimensions shaping the integration of environmental sustainability into chronic spinal pain care. Vertical coherence is needed to connect individual clinical reasoning with the institutional and policy levers that sustain it. Education, governance, and funding can embed sustainability as a professional standard, while equitable incentives and policy alignment support systemic transformation. The resulting framework bridges clinical, educational, and policy domains, linking planetary health, chronic pain, and professional responsibility to align clinical excellence with ecological and social accountability.

## Author Contributions

E.O. and N.K. drafted the initial protocol for securing funding. E.O. and A.M.M.P. refined the protocol, conducted literature reviews, and developed the methodology. They also constructed and formulated the statements for the Delphi questionnaire. E.O. and A.M.M.P. identified potential participants, compiled a list, and sent invitations to them. A.M.M.P. managed the e‐Delphi platform and ensured smooth operations throughout the study. E.O. and A.M.M.P. performed analyses between rounds and produced reports summarizing the results to adapt the statements for subsequent rounds, ensuring the iterative nature of the Delphi process was maintained. A.M.M.P. conducted statistical analyses to synthesize the results. E.O. wrote the initial draft of the manuscript, which was reviewed and revised by the other team members.

## Conflicts of Interest

The authors declare no conflicts of interest.

## Supporting information


**Methods S1:** Search strategies and eligibility criteria.
**Methods S2:** Round 1 e‐Delphi questionnaire.
**Methods S3:** Example of feedback provided to panelists.
**Results S1:** Clinical practice guideline recommendations and AGREE II ratings.
**Results S2:** Panelists' responses and priorities from the e‐Delphi study.
