# Supplementary material for: Environmental Physiotherapy for Chronic Spinal Pain Management: An International Modified Delphi Study
Source: Eur J Pain. 2026 Aug 11;30(7):e70354. doi: 10.1002/ejp.70354 (PMC13460240; doi:10.1002/ejp.70354)
Supplement: Supplementary file 1 [file EJP-30-0-s001.zip › methodsS1_R1.docx]

Supplementary Material

This supplementary material provides the terms used by our research team to find relevant clinical practice guidelines and literature about environmental sustainability in physiotherapy.

# 1 Systematic review of chronic spinal pain guidelines

The search terms to find clinical practice guidelines included the following terms:

- guideline or practice guideline or consensus development conference or consensus development conference, NIH
- guideline* or standards or consensus* or recommendat*
- practice parameter* or position statement* or policy statement* or CPG or CPGs or best practice*
- care adj2 (path or paths or pathway or pathways or map or maps or plan or plans or standard))
- (critical or clinical or practice) adj2 (path or paths or pathway or pathways or protocol*)
- algorithm* and (pharmacotherap* or chemotherap* or chemotreatment* or therap* or treatment* or intervention*)
- algorithm* and (screening or examination or test or tested or testing or assessment* or diagnosis or diagnoses or diagnosed or diagnosing)
- guideline* or standards or consensus* or recommendat*
- systematic review and (practice guideline* or treatment guideline* or clinical guideline* or guideline recommendation*)
- Chronic Pain/
- chronic adj2 pain*
- chronic adj2 (discomfort or ache*)
- persistent adj2 pain*

Eligibility criteria for the preliminary review of clinical practice guidelines

The preliminary review included evidence-based clinical practice guidelines (CPGs) addressing the conservative non-pharmacological management of chronic spinal pain in adults. Eligibility criteria were defined according to the population, scope of recommendations, document type, context, and publication characteristics.

Population. Eligible guidelines focus on adults aged 18 years or older living with chronic spinal pain. Chronic pain was defined as pain persisting or recurring for more than three months. Guidelines were excluded if they focused primarily on chronic central neuropathic pain, visceral pain, headache or orofacial pain, cancer-related pain, primary limb pain, widespread pain, or HIV-related pain.

Scope of recommendations. Eligible guidelines included recommendations related to assessment and evaluation, conservative non-pharmacological interventions, combined approaches to care, prevention, rehabilitation, education, exercise, or self-management. Guidelines focusing exclusively or primarily on medication alone, interventional management, such as injections or other invasive procedures, or surgery alone were excluded.

Type of document. Only evidence-based clinical practice guidelines were included. Consensus statements, position statements, practice alerts, narrative reviews, systematic reviews without guideline recommendations, and primary research studies were excluded.

Comparator. No comparator was required, as the review focused on clinical practice guidelines rather than intervention effectiveness studies.

Context. Guidelines from any country or healthcare context were eligible, provided that they addressed chronic spinal pain management in adults.

Outcomes. No primary clinical outcome was prespecified as an eligibility criterion, because the purpose of the review was to identify and synthesize guideline recommendations. Eligible CPGs were critically appraised using AGREE II, and recommendations were extracted to support the development of the Delphi statements.

Publication characteristics. Guidelines published between January 2018 and November 2023 were eligible. No language restrictions were applied.

# 2 Environmental impact of physical therapy treatments

The search strategy to find literature intersection environmental sustainability and physiotherapy included the following terms:

- environmental health or exp Environmental Health
- environmental monitoring or exp Environmental Monitoring
- environmental impact
- ecological sustainability
- exp "Conservation of Natural Resources"
- exp Environmental Pollution/ or exp Climate Change
- life cycle assessment
- carbon footprint or exp Carbon Footprint
- ecological footprint
- exp Medical Waste
- exp Recycling
- Physical Therapists
- physical therap*
- physiotherap*
- Physical Medicine and Rehabilitation
