# Supplementary material for: Environmental Physiotherapy for Chronic Spinal Pain Management: An International Modified Delphi Study
Source: Eur J Pain. 2026 Aug 11;30(7):e70354. doi: 10.1002/ejp.70354 (PMC13460240; doi:10.1002/ejp.70354)
Supplement: Supplementary file 1 [file EJP-30-0-s001.zip › methodsS2.docx]

Supplementary Material

This supplementary material presents all the statements and questions provided to the panelist on Round 1.

# 1 Participation Consent

By providing my consent, I confirm the following:

- I have read the Participation Information Sheet and have been given the opportunity to discuss about the process.

- I understand that this modified Delphi process seeks anonymity, and I agree not to disclose my identity in the comments to ensure the integrity of the data across rounds.

- I have been fully informed about the procedures and time commitment involved, and all my questions have been satisfactorily answered.

- I acknowledge that my participation in this Delphi process is voluntary, and I am not under any obligation to consent.

- I understand that I have the right to withdraw from the Delphi process at any time, without needing to provide a reason and without facing any consequences. However, I understand that the success of this study depends on completing all Delphi rounds.

- I know that my personal data only can be transmitted for research purposes in coded form. The promoter ensures data protection in accordance with Swiss standards and requirements.

- I am aware that the obligations mentioned in the Participant Information Sheet must be respected throughout the duration of the project.

Please note that the statements are written in American English. For "physiotherapy" or "physical therapy," the term "physical therapy" is used.

# 2 Background Information

How do you identify?

Year of birth:

Which of the following best describes your professional role? Choose the one that most accurately represents you.

Which of the following best describes the spinal region you work with most frequently?

In which country do you predominantly work? Please, insert the name in English.

Current education level:

In which year did you obtain your physical therapy (physiotherapy) degree?

Could you please indicate your level of interest in Environmental Sustainability and/or Ecology?

# 3 A. Environmental advocacy and policy

A.1. Government health departments or other regulatory health bodies should establish national guidelines encouraging sustainable healthcare practices in the management of chronic spinal pain.

A.2. Physical therapy organizations should create alliances and formulate policies with stakeholders at multiple levels (global, national, and local) to sustainably address chronic spinal pain.

A.3. Physical therapy organizations should demonstrate to policymakers and stakeholders the physical, economic, and environmental benefits of safe active transport infrastructure (e.g., bicycle lanes) for people with chronic spinal pain.

A.4. Physical therapists should actively incorporate the Sustainable Development Goals into their research to enhance the environmental and societal impact of physical therapy for chronic spinal pain management.

A.5. Research funding agencies should insist on sustainability as a key requirement for granting chronic spinal pain research.

A.6. Physical therapists involved in chronic spinal pain care should prioritize purchasing products from environmentally sustainable manufacturing processes.

A.7. Physical therapists involved in chronic spinal pain care should exert pressure on suppliers to minimize packaging and adopt energy-efficient equipment.

A.8. Healthcare administrators should adopt policies that incentivize environmentally friendly physical therapy practices for chronic spinal pain management.

A.9. Please suggest new statements or topics about "Environmental advocacy and policy" not yet addressed that you believe should reach a consensus.

# 4 B. Education and professional development

B.1. Physical therapists should regularly consult and integrate clinical practice guidelines for chronic spinal pain that incorporate environmental impact considerations into all aspects of patient care.

B.2. Clinical practice guidelines for the management of chronic spinal pain should undergo regular review to ensure alignment with the latest environmental standards and considerations in healthcare research.

B.3. Physical therapists should educate themselves and their healthcare colleagues to effectively integrate sustainable practices in managing chronic spinal pain.

B.4. Physical therapy education programs should integrate content on environmental health, including its relationship to colonialism, social justice, and health equity, into the management of chronic spinal pain conditions.

B.5. Physical therapy training should equip practitioners with the skills to utilize outdoor exercises and natural environments as effective treatment strategies for chronic spinal pain.

B.6. Alternative payment models should be established for remote consultations to ensure the financial stability of physical therapists involved in chronic spinal pain care.

B.7. Although physical therapy is often considered eco-friendly (using modalities such as movement, communication, and touch), physical therapists should further educate themselves about sustainable practices for managing chronic spinal pain conditions.

B.8. Continuing education courses should provide training in sustainable approaches for managing chronic spinal pain to ensure that physical therapists remain up to date on these evolving practices after graduation.

B.9. Physical therapists should incorporate environmental considerations into their clinical reasoning when managing chronic spinal pain.

B.10. Physical therapists should learn to use Generative Artificial Intelligence (e.g., ChatGPT, Gemini, Copilot…) for developing treatment plans that minimize environmental impact.

B.11. Please suggest new statements or topics about "Education and professional development" not yet addressed that you believe should reach a consensus.

# 5 C. Research and innovation in treatment methods

C.1. Supported by academic institutions and healthcare organizations, physical therapists should actively participate in empirical research studies to understand the environmental effects of treatments for chronic spinal pain.

C.2. Journal and review editors should mandate that all submitted research and review articles on the management of chronic spinal pain demonstrate the consideration of environmentally conscious practices as a condition for publication.

C.3. Researchers should systematically analyze both the therapeutic results and environmental impacts of physical therapy treatments, to inform clinical decision-making in chronic spinal pain management.

C.4. Researchers should conduct studies to evaluate the environmental impact and clinical efficacy of telehealth and digitalization in the management of chronic spinal pain, ensuring that both sustainability and treatment effectiveness are prioritized.

C.5. Physical therapists and researchers should develop comprehensive prevention strategies that address the interplay of biological, psychological, social, and environmental factors contributing to chronic spinal pain to reduce its occurrence and improve patient outcomes.

C.6. Researchers should measure and compare the environmental impacts of physical therapy treatments with those of other healthcare interventions for chronic spinal pain.

C.7. Researchers should explore the effectiveness, barriers, and enablers of nature-based interventions (e.g., outdoor exposure) in managing chronic spinal pain, to optimize their practical application and outcomes.

C.8. Physical therapists and researchers should develop a tool to estimate the environmental footprint of their physical therapy practices in chronic spinal pain (e.g., footprint calculator).

C.9. Researchers in chronic spinal pain care should conduct studies to assess whether the digitalization of physical therapy services offers a net positive impact on environmental sustainability.

C.10. Future research should investigate barriers within the physical therapy management of chronic spinal pain with a focus on embedding sustainability into all aspects of treatment protocols and clinical procedures.

C.11. Physical therapists should leverage Generative Artificial Intelligence (e.g., ChatGPT, Gemini, Copilot…) to innovate and implement green solutions in the management of chronic spinal pain.

C.12. Please suggest new statements or topics about "Research and innovation in treatment methods" not yet addressed that you believe should reach a consensus.

# 6 D. Community engagement and patient empowerment

D.1. Physical therapists involved in chronic spinal pain care should lead by example in adopting and promoting a culture of environmental sustainability within their practices and communities, as part of a broader effort to mitigate climate change.

D.2. Physical therapists should establish partnerships with individuals with chronic spinal pain to jointly discuss and select treatments that balance both individual needs and environmental concerns, adapting these decisions to the specific contexts and circumstances of the treatment.

D.3. Physical therapy should be promoted as a sustainable treatment solution for chronic spinal pain by reducing reliance on imaging and medication.

D.4. Physical therapists should incorporate nature-based interventions (e.g., exposure to green and blue spaces) for treating patients with chronic spinal pain conditions.

D.5. Physical therapists should develop a comprehensive understanding of global environmental issues and their effects on patients with chronic spinal pain, enabling them to collaboratively and appropriately tailor treatment decisions with their patients.

D.6. Physical therapists should integrate traditional and Indigenous knowledge to enhance holistic management of chronic spinal pain, aligning environmental, health, and community factors.

D.7. Physical therapists, researchers, and interprofessional teams should develop care plans in partnership with patients, focusing on self-efficacy and self-management to empower individuals with chronic spinal pain.

D.8. Physical therapists involved in chronic spinal pain care should play a role in community projects that encourage physical activity and ecological restoration (e.g., public health strategies, urban planning, etc.).

D.9. Physical therapists should educate patients with chronic spinal pain about the health and environmental benefits of active transport (e.g., cycling) and outdoor exposure (e.g., hiking).

D.10. Patient advocacy groups should collaborate with physical therapists and researchers to identify barriers and enablers to implementing environmentally sustainable actions for managing chronic spinal pain.

D.11. Non-governmental organizations focused on the environment and health should raise awareness about the importance of sustainable physical therapy practices in managing chronic spinal pain.

D.12. Please suggest new statements or topics about "Community engagement and patient empowerment" not yet addressed that you believe should reach a consensus.

# 7 E. Sustainable clinical practices

E.1. Physical therapists should adopt the sustainable value equation as a guiding framework for decision-making in chronic spinal pain care.

Figure 1. Sustainable value in health care. Retrieved from: *Palstam, A., Andersson, M., Lange, E., & Grenholm, A. (2021). A Call to Include a Perspective of Sustainable Development in Physical Therapy Research. Physical therapy, 101(3), pzaa228. https://doi.org/10.1093/ptj/pzaa228*

This approach ensures that treatment outcomes are not only clinically effective but also environmentally sustainable and socially responsible.

E.2. Physical therapists should actively minimize overuse and unnecessary procedures in the management of chronic spinal pain (e.g., considering the principles of initiatives like “Smarter Medicine” or “Choosing Wisely”).

E.3. Physical therapists involved in chronic spinal pain care should identify and implement basic sustainable practices (e.g., reducing electricity consumption, minimizing waste, etc.).

E.4. Physical therapists should explore and integrate digital health tools (e.g., remote consultation systems, apps…) in the management of chronic spinal pain conditions to reduce time, financial, and environmental costs, while ensuring such tools also support favorable patient outcomes and maintain clinical effectiveness.

E.5. Physical therapists involved in chronic spinal pain care should promote and use active transport, such as cycling or walking, whenever possible, thereby offering co-benefits of environmental sustainability and improved personal well-being.

E.6. Technology developers should ensure that digital health solutions for chronic spinal pain management are designed with eco-friendly features and minimal electronic footprints.

E.7. When exploring the potential of Generative Artificial Intelligence (e.g., ChatGPT, Gemini, Copilot…) for the management of chronic spinal pain, physical therapists should be aware of its environmental impact and make efforts to utilize it responsibly (e.g., by minimizing unnecessary prompts).

E.8. Please suggest new statements or topics about "Sustainable clinical practices" not yet addressed that you believe should reach a consensus.

# 8 F. Priorities

F.1. In the context of managing chronic spinal pain and promoting environmental sustainability, what should physical therapists prioritize in terms of time and effort? Please order the following items:

# 9 G. Final thoughts

G.1. Please suggest new statements or topics not addressed in this modified Delphi that you believe should reach a consensus.

# 10 End

Thank you for your valuable time and insights! Your participation in this Delphi study is greatly appreciated. We will be compiling the results and sending out a follow-up survey for further refinement.
