# Supplementary material for: Environmental Physiotherapy for Chronic Spinal Pain Management: An International Modified Delphi Study
Source: Eur J Pain. 2026 Aug 11;30(7):e70354. doi: 10.1002/ejp.70354 (PMC13460240; doi:10.1002/ejp.70354)
Supplement: Supplementary file 1 [file EJP-30-0-s001.zip › methodsS3.docx]

Supplementary Material

# 1 Example of feedback provided to panelists

This example shows the modifications of one statement between Round 1 and Round 2.

The graph illustrates the relationship between participants' level of agreement and the degree of importance they assign to the statement in Round 1. The X-axis represents the level of agreement, ranging from "Strongly Disagree" to "Strongly Agree," while the Y-axis shows the degree of importance, from "Not at All Important" to "Extremely Important." Each bubble's size corresponds to the number of participants who selected a particular combination of agreement and importance.

Below the graph, the median level of agreement is provided, along with its interquartile range. Similarly, the median degree of importance is presented with its corresponding interquartile range.

The median, a measure of central tendency, is the middle value of a dataset when the numbers are arranged in ascending or descending order. In this context, a median of 1 indicates that most participants leaned toward disagreement or assigning low importance, while medians of 2, 3, or 4 reflect increasingly positive responses, with higher values indicating a trend toward agreement or perceiving higher importance.

The interquartile range (IQR) measures the spread of the middle 50% of the dataset. It is calculated as the difference between the third quartile (Q3, the 75th percentile) and the first quartile (Q1, the 25th percentile): IQR = Q3 − Q1. An IQR of 1 suggests low variability, indicating that participants generally provided similar responses, while IQRs of 2, 3, or 4 reflect increasing levels of variability, with higher values indicating more diverse responses among participants.

Below the quantitative measures (median and degree of importance), the participants' comments are also provided.

Finally, we provided the proposed modifications in red, based on the participants’ feedback.

| D.4. Physical therapists should incorporate nature-based interventions (e.g., exposure to green and blue spaces) for treating patients with chronic spinal pain conditions. | |
| --- | --- |
|  | |
| Level of agreement   - Median = 4 - Interquartile range = 1 | Degree of importance   - Median = 4 - Interquartile range = 2 |
| Comments:   1. It can be explored. 2. If the evidence supports it, why not 3. Only after the research to support the practice 4. Definitely! And let's communicate more on the effects of these exposures to patients, colleagues, etc.! 5. Absolutely, but there isn't evidence yet for these interventions for pain (although it is great for general well-being!) 6. Only as part of patient-centred care 7. If evidence-based and fit individual patient preferences 8. Improves mental health as well and is often less of a financial burden. | |
| Modifications:  D.4.1. Physical therapists should consider incorporating nature-based interventions (e.g., exposure to green and blue spaces) for treating patients with chronic spinal pain conditions, provided these approaches are evidence-based and aligned with individual patient preferences. | |
