# Supplementary material for: Environmental Physiotherapy for Chronic Spinal Pain Management: An International Modified Delphi Study
Source: Eur J Pain. 2026 Aug 11;30(7):e70354. doi: 10.1002/ejp.70354 (PMC13460240; doi:10.1002/ejp.70354)
Supplement: Supplementary file 1 [file EJP-30-0-s001.zip › resultsS1_R1.docx]

Supplementary Material

# 1 Guideline quality assessment according to the AGREE-II instrument

Quality rating according to Johnston et al. (2018):

| Criterion | Quality Rating |
| --- | --- |
| Individual domain scores |  |
| ≥ 60% | Adequately or effectively addressed |
| Domain scores |  |
| ≥ 60% in at least 3 domains, including domain 3 | High quality |
| ≥ 60% of 3 or more domains, except domain 3 | Moderate quality |
| ≥ 60% in at least 2 domains, domain 3 > 50 | Moderate quality |
| All other scores | Low quality |

Results of the quality assessment:

| Guideline | AGREE-II Domain | | | | | | | Quality | Included |
| --- | --- | --- | --- | --- | --- | --- | --- | --- | --- |
|  | Target Region | Scope and purpose | Stakeholder involvement | Rigour of development | Clarity and presentation | Applicability | Editorial independence |  |  |
| ACOEM (2019)^1^ | Lower back | 83% | 61% | 79% | 83% | 42% | 50% | High | Yes |
| BASGK (2019)^2^ | Lower back | 83% | 56% | 54% | 89% | 25% | 67% | Moderate | No |
| Bussières et al. (2018)^3^ | Lower back | 94% | 78% | 69% | 83% | 63% | 75% | High | Yes |
| CDWC (2022)^4^ | Neck | 78% | 39% | 65% | 89% | 21% | 33% | High | Yes |
| CDWC (2022)^5^ | Lower back | 78% | 44% | 65% | 83% | 17% | 25% | High | Yes |
| Colvin et al. (2019)^6^ | Neck and lower back | 94% | 83% | 83% | 94% | 67% | 92% | High | Yes |
| VADoD (2022)^7^ | Lower back | 94% | 89% | 83% | 94% | 58% | 58% | High | Yes |
| George et al. (2021)^8^ | Lower back | 100% | 78% | 67% | 100% | 29% | 75% | High | Yes |
| Korownk et al. (2022)^9^ | Lower back | 72% | 94% | 73% | 78% | 42% | 75% | High | Yes |
| NICE (2020)^10^ | Lower back | 94% | 83% | 92% | 89% | 75% | 83% | High | Yes |
| NICE (2021)^11^ | Neck | 83% | 83% | 85% | 83% | 71% | 75% | High | Yes |
| NASS (2020)^12^ | Lower back | 100% | 78% | 88% | 94% | 33% | 75% | High | Yes |
| JSAM et al. (2021)^13^ | Neck and Lower back | 56% | 67% | 58% | 61% | 21% | 33% | Moderate | No |
| WHO (2023)^14^ | Lower back | 100% | 94% | 85% | 94% | 46% | 100% | High | Yes |

# 2 Global comparison of Clinical Practice Guidelines recommendations for lumbar or lower back region

| **Key recommendations** | **ACOEM^1^** | **Bussieres^3^** | **CDLE^5^** | **George^8^** | **NASS^12^** | **NICE^10^** | **Korownyk^9^** | **Colvin^6^** | **VA/DoD^7^** | **WHO^14^** |
| --- | --- | --- | --- | --- | --- | --- | --- | --- | --- | --- |
| **EDUCATION AND ADVICE** | | | | | | | | | | |
| Standard education strategies OR Structured education OR Clinician-directed education with patient-led goal setting OR Patient education OR Brief education -> as intervention OR as adjunct |  |  | Moderate | Moderate | Very low |  |  | Low | Very low | Very low |
| Pain neuroscience education -> as adjunct |  |  | Moderate | High |  |  |  |  | Very low |  |
| Advice to stay active OR Advice interventions -> as adjunct |  | High |  |  |  |  |  | High |  | Very low |
| Bed rest | Moderate |  | Moderate |  |  |  |  |  |  |  |
| Referral to a pain management programme |  |  |  |  |  |  |  | Low |  |  |
| Specific beds or other commercial sleep products | Very low |  |  |  |  |  |  |  |  |  |
| Lordotic sitting posture | Low |  |  |  |  |  |  |  |  |  |
| Back school |  |  |  |  | High |  |  |  | Very low |  |
| Self-management OR Self help resources OR Addition of supplementary material -> as adjunct |  |  |  |  |  | Low |  | Low |  |  |
| Sleep posture adjustment | Very low |  |  |  |  |  |  |  |  |  |
| Mattresses, water beds, and other sleeping surfaces | Very low |  |  |  |  |  |  |  |  |  |
| Changing sleeping surfaces | Very low |  |  |  |  |  |  |  |  |  |
| Belts or corsets OR Lumbar supports OR Bracets | Low |  |  |  |  | Low |  |  | Very low | Very low |
| Shoe insoles and lifts OR Foot orthotics OR Rocker sole shoes | Very low |  |  |  |  | Low |  |  |  |  |
| **PHYSICAL ACTIVITY** | | | | | | | | | | |
| Exercise prescription OR Exercise training interventions OR General exercise OR Physical activity OR Exercise therapy OR Structured exercise programme OR Supervised exercise sessions | Moderate |  | Moderate | High |  |  | Moderate | Moderate |  | Low |
| Slump stretching OR Neural mobilization -> as adjunct or short term | Very low |  |  | Moderate |  |  |  |  |  |  |
| Work hardening or conditioning |  |  |  |  | Low |  |  |  |  |  |
| Stretching exercises | Very low |  |  | High |  |  |  |  |  |  |
| Strengthening exercises OR Trunk muscle strengthening and endurance | Low |  |  | High |  |  |  |  | Very low |  |
| Aerobic exercise | High |  |  | High | High | Low |  |  | Very low |  |
| Directional exercise | Very low |  |  |  |  |  |  |  |  |  |
| Aquatic therapy | Low |  |  | High |  |  |  |  | Very low |  |
| Lumbar extension machines | Very low |  |  |  |  |  |  |  |  |  |
| Biomechanical |  |  |  |  |  | Low |  |  |  |  |
| Mobility exercises |  |  |  | Moderate |  |  |  |  | Very low |  |
| Yoga | Low |  | Moderate | High | Moderate |  |  |  | Low |  |
| Qi gong |  |  |  |  |  |  |  |  | Very low |  |
| Tai chi | Low |  | Low |  |  |  |  |  | Very low |  |
| Pilates | Very low |  | Moderate | High |  |  |  |  | Very low |  |
| Movement control exercise OR Motor control |  |  | Moderate | Moderate |  |  |  |  | Very low |  |
| Specific trunk muscle activation exercise OR Active stabilization exercise OR Lumbar stabilization | Low |  |  | High | Very low |  |  |  |  |  |
| Structured walking program |  |  |  |  |  |  |  |  | Very low |  |
| Multimodal exercise interventions |  |  |  | High |  |  |  |  |  |  |
| Wearable activity trackers OR Technology-based modalities |  |  |  |  | Very low |  | Very low |  | Very low |  |
| EMG biofeedback OR Biofeedback as adjunct |  |  | Moderate |  |  |  |  | Low |  |  |
| McKenzie method OR Mechanical diagnosis and therapy |  |  | Moderate |  | Low |  |  |  | Very low |  |
| Group exercise program OR Combined group |  |  |  |  |  | Low |  | Moderate |  |  |
| Home exercise OR Home exercise programme-> as adjunct |  |  |  |  | Very low |  |  | Moderate |  |  |
| **PHYSICAL AGENTS AND MODALITIES** | | | | | | | | | | |
| Inversion therapy | Very low |  |  |  |  |  |  |  |  |  |
| Kinesiotaping (Including KT Tape and Rocktape) and taping | Low |  | Very low |  |  |  |  |  |  |  |
| Dry needling OR Needling therapies |  |  |  | Low | Very low |  |  |  |  | Low |
| Magnets | Moderate |  |  |  |  |  |  |  |  |  |
| Cupping |  |  |  |  |  |  |  |  | Low |  |
| Hyperbaric Oxygen | Very low |  |  |  |  |  |  |  |  |  |
| Iontophoresis | Very low |  | Very low |  |  |  |  |  |  |  |
| Phonophoresis |  |  | Very low |  |  |  |  |  |  |  |
| Cryotherapies (may be tried) | Very low |  |  |  |  |  |  |  |  |  |
| Heat therapies | Low |  |  |  |  |  |  |  |  |  |
| Diathermy | Low |  |  |  |  |  |  |  |  |  |
| Infrared Therapy | Very low |  |  |  |  |  |  |  |  |  |
| Ultrasound OR Therapeutic ultrasound | Very low |  | Very low |  | Very low | Very low |  |  | Low | Low |
| Low-level laser therapy OR Laser therapy OR Laser therapy as adjunct | Low |  | Very low |  | Moderate |  |  | Moderate | Low |  |
| Laser acupuncture |  |  | Moderate |  | High |  |  |  |  |  |
| Interferential therapy | Very low |  |  |  |  | High |  |  |  |  |
| Transcutaneous Electrical Neurostimulation (TENS) -> as adjunct or treatment | Very low |  |  |  | Very low | Very low |  | Moderate | Low | Very low |
| Neromuscular Electrical Stimulation (NMES) as adjunct | Very low |  |  |  |  |  |  |  |  |  |
| Percutaneous Electrical Nerve Stimulation (PENS) | Very low |  | Moderate |  |  | Low |  |  |  |  |
| Microcurrent electrical stimulation | Very low |  |  |  |  |  |  |  |  |  |
| H-Wave Device stimulation | Very low |  |  |  |  |  |  |  |  |  |
| High-voltage galvanic therapy | Very low |  |  |  |  |  |  |  |  |  |
| **MANUAL INTERVENTIONS** | | | | | | | | | | |
| Massage OR Soft tissue mobilization OR Passive therapies -> as adjunct or short term or treatment | Low |  | Moderate | Moderate | Very low | Very low |  |  |  | Very low |
| Myofascial release OR Myofascial therapy (as adjunt) | Very low | High |  |  |  |  |  |  |  |  |
| Traction (including Decompression through traction and spinal decompressive devices OR Mechanical traction OR Mechanical lumbar traction) | High |  | Moderate | Very low | High | High |  |  | Low | Very low |
| Spinal Manipulative Therapy (SMT) OR Spinal manipulation |  | Very low |  |  | Low |  | Low |  |  | Very low |
| Manipulation or mobilization OR Thrust or nonthrust joint mobilization OR Manual therapy-> for short term or as adjunct | Low |  | Moderate | High |  | High |  | Moderate | Low |  |
| Manipulation Under Anesthesia (MUA) and Medication-Assisted Spinal Manipulation (MASM) | Very low |  |  |  |  |  |  |  |  |  |
| **PSYCHOLOGICAL INTERVENTIONS** | | | | | | | | | | |
| Cognitive behavioral therapy OR Cognitive behavioural approach OR Cognitive therapy -> as adjunct or treatment |  |  | Moderate |  | Very low | Low | Low | Low | Low | Very low |
| Operant therapy |  |  |  |  |  |  |  |  |  | Very low |
| Respondent therapy |  |  |  |  |  |  |  |  |  | Very low |
| **ALTERNATIVE AND COMPLEMENTARY METHODS** | | | | | | | | | | |
| Complementary or alternative methods or dietary supplements OR Specific diet or nutritional, herbal, or homeopathic supplements | Very low |  |  |  |  |  |  |  | Very low |  |
| Medical foods | Very low |  |  |  |  |  |  |  |  |  |
| Herbal and other preparations | Very low |  |  |  |  |  |  |  |  |  |
| Reflexology | Low |  |  |  |  |  |  |  |  |  |
| Auricular acupressure |  |  |  |  |  |  |  |  | Low |  |
| Acupressure |  |  |  |  | Very low |  |  |  |  |  |
| Neroreflexotherapy as alternative | Low |  |  |  |  |  |  |  |  |  |
| Acupuncture -> as adjuct or treatment | Low |  | Moderate |  | High | Moderate | Very low | High | Low | Low |
| Mindfulness-based stress reduction OR Progressive relaxation |  |  | Moderate |  |  |  | Low | Low | Low | Low |
| **MULTIMODAL INTERVENTIONS** | | | | | | | | | | |
| Combined physical and psychological programme OR Multidisciplinary or interdisciplinary program OR Multicomponent biopsychosocial care |  |  |  |  |  | Low |  |  | Very low | Low |
| Multimodal therapy with or without SMT |  | Moderate |  |  |  |  |  |  |  |  |
| SMT plus home exercise and advice |  | Low |  |  |  |  |  |  |  |  |
| Non-pharmacological weight loss |  |  |  |  |  |  |  |  |  | Very low |
| Physical and Occupational therapy | Very low |  |  |  |  |  |  |  |  |  |

GRADE – quality of evidence:

Very low / Low / Moderate / High

# 3 Global comparison of Clinical Practice Guidelines recommendations for cervical or neck region

| **Key recommendations** | **CDLE^4^** | **NICE^11^** | **Colvin^6^** |
| --- | --- | --- | --- |
| **EDUCATION AND ADVICE** |  |  |  |
| Advice and information (including self-management) OR Self-management resources OR Self-help resources OR Addition of supplementary material |  | Moderate |  |
| Encourage to maitain physical activity OR Advice to stay active OR Brief education OR Patient education | Low | Moderate |  |
| **PHYSICAL ACTIVITY** |  |  |  |
| Therapeutic exercise OR Exercise and exercise therapies OR Active therapy program OR Supervised exercise sessions | Moderate |  | Moderate |
| Functional treatment | Moderate |  |  |
| Supervised group exercise programme OR Individualised exercises in group settings |  | Low | Moderate |
| Biofeedback OR EMG biofeedback | Moderate |  |  |
| Home exercise programme |  |  | Moderate |
| McKenzie | Low |  |  |
| Pilates | Moderate |  |  |
| Tai Chi | Low |  |  |
| **PHYSICAL AGENTS AND MODALITIES** |  |  |  |
| Mechanical traction OR Motorized traction | Low |  |  |
| Ultrasound | Very low | Very Low |  |
| TENS OR Transcutaneous electrical nerve stimulation |  | Very Low | Moderate |
| Phonophoresis | Very low |  |  |
| Low level laser | Very low |  |  |
| Interferential therapy |  | Very Low |  |
| Kinesiotaping | Very low |  |  |
| Iontophoresis | Very low |  |  |
| **MANUAL INTERVENTIONS** |  |  |  |
| Passive therapies - as adjunct | Moderate |  |  |
| Manual therapy (in combination with exercise) |  |  | Moderate |
| Manipulation and mobilization | Moderate |  |  |
| Massage | Low |  |  |
| Craniosacral Therapy | Low |  |  |
| **ALTERNATIVE AND COMPLEMENTARY METHODS** |  |  |  |
| Acupuncture (one try) | Moderate | Very Low |  |
| Dry needling (one try) | Low | Very Low |  |
| Progressive relaxation OR Relaxation Training | Moderate |  |  |
| **PSYCHOLOGICAL INTERVENTIONS** |  |  |  |
| Acceptance and commitment therapy (ACT) |  | Very Low |  |
| CBT OR Cognitive behavioural therapy | Moderate |  |  |

GRADE – quality of evidence:

Very low / Low / Moderate / High

References

Johnston A, Hsieh SC, Carrier M, et al. A systematic review of clinical practice guidelines on the use of low molecular weight heparin and fondaparinux for the treatment and prevention of venous thromboembolism: Implications for research and policy decision-making. *PLoS One* 2018; 13: e0207410. DOI: 10.1371/journal.pone.0207410.

# Clinical Practice Guidelines:

1.	American College of Occupational and Environmental Medicine. ACOEM Practice Guidelines: Low Back Disorders. 2019.

2.	Bundesministerium für Arbeit Soziales Gesundheit und Konsumentenschutz. Update der evidenz- und konsensbasierten Österreichischen Leitlinie für das Management akuter, subakuter, chronischer und rezidivierender unspezifischer Kreuzschmerzen 2018 – Kurzbezeichnung Leitlinie Kreuzschmerz 2018. 2018.

3.	Bussieres AE, Stewart G, Al-Zoubi F, et al. Spinal manipulative therapy and other conservative treatments for low back pain: A guideline from the Canadian chiropractic guideline initiative. *Journal of Manipulative and Physiological Therapeutics* 2018; 41: 265–293. DOI: 10.1016/j.jmpt.2017.12.004.

4.	Colorado Division of Workers' Compensation. Cervical spine injury medical treatment guidelines. In: Colorado Department of Labor and Employment, (ed.). 2021.

5.	Colorado Division of Workers' Compensation. Low Back Pain Medical Treatment Guidelines. In: Colorado Department of Labor and Employment, (ed.). 2021.

6.	Colvin L, Atherton R, Bannister J, et al. Management of chronic pain: a national clinical guideline (Scottish Intercollegiate Guidelines Network SIGN136). 2019.

7.	Department of Veterans Affairs / Department of Defense. VA/DoD clinical practice guideline: The diagnosis and treatment of low back pain. Washington DC: U.S. Government Printing Office,, 2022.

8.	George SZ, Fritz JM, Silfies SP, et al. Interventions for the Management of Acute and Chronic Low Back Pain: Revision 2021. *J Orthop Sports Phys Ther* 2021; 51: CPG1–CPG60. 2021/11/02. DOI: 10.2519/jospt.2021.0304.

9.	Korownyk CS, Montgomery L, Young J, et al. PEER simplified chronic pain guideline: Management of chronic low back, osteoarthritic, and neuropathic pain in primary care. *Canadian family physician Medecin de famille canadien* 2022; 68: 179–190. 2022/03/17. DOI: 10.46747/cfp.6803179.

10.	National Institute for Health and Care Excellence. Low back pain and sciatica in over 16s: assessment and management (NICE guideline NG59). 2020.

11.	National Institute for Health and Care Excellence. Chronic pain (primary and secondary) in over 16s: assessment of all chronic pain and management of chronic primary pain (NICE Guideline NG193). 2021.

12.	North American Spine Society. Evidence-based clinical guidelines for multidisciplinary spine care: Diagnosis & treatment of low back pain. 2020.

13.	The Japan Society of Acupuncture and Moxibustion, Japanese Association for the Study of Musculoskeletal Pain, Japanese Society of Orofacial Pain, et al. Clinical practice guidelines for the management of chronic pain. Publication Department, Shinko Trading Co. Ltd., 2021.

14.	World Health Organization. WHO guideline for non-surgical management of chronic primary low back pain in adults in primary and community care settings. Geneva. Licence: CC BY-NC-SA 3.0 IGO, 2023.
