# Supplementary material for: Environmental Physiotherapy for Chronic Spinal Pain Management: An International Modified Delphi Study
Source: Eur J Pain. 2026 Aug 11;30(7):e70354. doi: 10.1002/ejp.70354 (PMC13460240; doi:10.1002/ejp.70354)
Supplement: Supplementary file 1 [file EJP-30-0-s001.zip › resultsS2.docx]

Supplementary Material

This supplementary material provides detailed results of the multiple-selection questions, ordering questions, and Yes/No/I don’t know questions. Note that intermediate questions, which were adapted or reformulated, are not presented here. Only the final results of non-statement questions are included.

# 1 ROUND 2

## 1.1 Multiple-selection questions

### 1.1.1 What environmental health contents should be integrated into physical therapy education programs?

### 1.1.2 Which topics should physical therapists address to raise environmental health awareness of patients with chronic spinal pain?

### 1.1.3 To ensure alignment with the latest environmental research in healthcare, what specific environmental considerations should be included in clinical practice guidelines for chronic spinal pain management?

## 1.2 Ordering questions

### 1.2.1 In the context of managing chronic spinal pain and promoting environmental sustainability, what should physical therapists prioritize in terms of time and effort within “A. Environmental advocacy and policy”?

### 1.2.2 Which strategies or formats for rapid knowledge dissemination would you recommend to promote education and professional development of physical therapists on environmental sustainability?

### 1.2.3 In the context of managing chronic spinal pain and promoting environmental sustainability, what should physical therapists prioritize in terms of time and effort within “B. Education and professional development”?

### 1.2.4 In the context of managing chronic spinal pain and promoting environmental sustainability, what should physical therapists prioritize in terms of time and effort within “C. Research and innovation in treatment methods”?

### 1.2.5 In the context of managing chronic spinal pain and promoting environmental sustainability, what should physical therapists prioritize in terms of time and effort within “D. Community engagement and patient empowerment”?

### 1.2.6 In the context of managing chronic spinal pain and promoting environmental sustainability, what should physical therapists prioritize in terms of time and effort within “E. Sustainable clinical practices”?

## 1.3 Question Yes/No/I don’t know

### 1.3.1 Is it appropriate for physical therapists to raise awareness about environmental impacts of treatments (from physical therapy or others) among patients who appear unconcerned about such matters?

# 2 ROUND 3

## 2.1 Ordering question

### 2.1.1 In the context of integrating environmental awareness into healthcare, under what conditions should physical therapists engage patients in discussions about environmental issues?

## 2.2 Question Yes/No/I don’t know

### 2.2.1 Appropriateness of environmental sustainability in physical therapy
